# Molecular and transcriptional regulation of plant defense responses to aphid infestation

**DOI:** 10.1007/s44154-026-00336-y

**Published:** 2026-09-01

**Authors:** Vishal Patil, Rizwana Rehsawla, Apurba K. Barman

**Affiliations:** 1 Department of Entomology, College of Agricultural and Environmental Sciences, University of Georgia, Tifton, GA 31793 USA; 2 J.C. Bose University of Science and Technology, YMCA, Faridabad, Haryana 121006 India; 3 Department of Life Sciences, DPG Degree College, Gurugram, Haryana 122004 India

**Keywords:** Aphid, Plant defense, Systemic resistance, Hormonal regulation, Transcription factors, Sustainable pest management

## Abstract

Aphids are one of the important agricultural pests causing substantial yield losses in crops grown across the globe. Aphids are known to cause direct feeding damages and indirect losses due to sooty mold development and plant virus transmission. Plants respond to these attacks by mounting a complex defense response at the infested sites and systemic levels. This multilayered defense response involves a highly coordinated network of phytohormones and other signalling components like Ca^2+^, mitogen activated protein kinases and reactive oxygen species. Key to these complex responses is a well-regulated gene expression involving several transcription factors. A wide range of transcription factors are structurally and functionally characterized across some model plants and in a few agronomically important crops. These transcription factors play diverse roles such as defense gene expression modulation, regulation of hormone signaling, secondary metabolism, oxidative stress response, cell wall modifications, and phloem-based defense. Understanding the integration of signaling pathways, hormone crosstalk, and transcription factor mediated regulation provides a framework for practical applications, including breeding, genome editing, and elicitor-based strategies. This review highlights how plant defense signaling and transcriptional regulation against aphids can be harnessed to develop sustainable and novel pest management solutions.

## Introduction

Aphids are serious Global pests due to the extent of damage they cause to the wide range of economically important crops. They derive nutrition exclusively from the plant phloem sap using piercing-sucking mouthparts (Singh and Singh 2021; Alyokhin et al. 2022). Some aphids damage plants by extracting nutrients thereby altering the host physiology and morphology (Jakobs et al. 2019). They also facilitate the growth of sooty mold that hampers plant respiration and reduce photosynthesis (Singh and Singh 2021; Kumaraswamy and Huang 2024). Aphids like *Myzus persicae* alter the host transcriptional machinery and secondary metabolism in *Arabidopsis thaliana*, celery and other plants (Thompson and Goggin 2006). Importantly, they also serve as efficient vectors for numerous plant viruses and greatly amplify their negative impact on crop productivity (Guo et al. 2022; Jayasinghe et al. 2022; Warnasooriya et al. 2024).

During the co-evolution process, plants and aphids have developed a complex interaction leading to well-orchestrated plant defense mechanisms against aphids. This involves PAMP-triggered immunity (PTI), effector-triggered immunity (ETI), and systemic immune responses like systemic acquired resistance (SAR) and Induced Systemic Resistance (ISR) (Moran and Thompson 2001; Jaouannet et al. 2014). Phytohormone-mediated defense activation and synthesis of secondary metabolites such as plant phenolics, flavonoids, tannins, lectins and protease inhibitors are key components of plant- aphid interactions (Kuśnierczyk et al. 2008; Arimura et al. 2009; War et al. 2012; Züst and Agrawal 2016; Kumaraswamy and Huang 2024; Yang et al. 2024; Gebretsadik et al. 2025). Additionally, herbivore-induced plant volatiles (HIPVs), premature leaf chlorosis and hypersensitive cell death are another crucial aspect of the plant defense against these insects (Pegadaraju et al. 2005; Arimura et al. 2009). Central to these defense processes is the tight regulation of gene expression, which governs the plant’s ability to perceive aphid attack and initiate appropriate responses. Aphids, in turn, manipulate host defense responses through the secretion of several effector proteins delivered via their saliva once their stylets enter the apoplast and subsequently the phloem (Rodriguez and Bos 2013). Aphid salivary components suppress sieve element occlusion and circumvent multiple layers of plant defense (Thompson and Goggin 2006; Goggin 2007; Will et al. 2007; Walling 2008). Some of these effectors including homologs of Macrophage Migration Inhibition Factor (MIFs), Cathepsin B (CathB), and C002, aids the aphids by suppressing the host defense response (Naessens et al. 2015; Reymond and Calandra 2015; Xue et al. 2024; Liu et al. 2025). Whereas numerous others like Cathepsin B3 (CathB3), SmCSP4, MP10 act as an elicitor for plant defense activation (Guo et al. 2020; Zhang et al. 2023a; Gravino et al. 2025).

Transcriptomic studies across diverse plant-aphid systems have revealed that aphid colonization induces extensive reprogramming of the host transcriptional landscape (Huang and Huang 2023). Importantly, Aphids such as *M. persicae, Macrosiphoniella sanbourni, Macrosiphum euphorbiae Thomas,* and *Aphis gossypii Glover,* feeding modulates systemic expression of thousands of genes, which is far greater than the localized and comparatively modest transcriptional responses elicited by chewing herbivores in plants like *A. thaliana, Chrysanthemum morifolium*, tomato and cotton (Appel et al. 2014; Xia et al. 2014; Huang and Huang 2023, Fernandez Martin and Reymond 2025). This highlights the specialized molecular strategies employed by plants to perceive and respond to phloem-feeding herbivores. Moreover, Aphids themselves undergo transcriptional changes in response to host quality and environmental conditions, further emphasizing the dynamic molecular interactions between plant and insect (Le Trionnaire et al. 2013; Matsuda et al. 2020; Thorpe et al. 2020). *Myzus cerasi* (black cherry aphid) undergo extensive transcriptional changes to changing environments, when moved from its primary host i.e. cherry trees to the secondary host, land cress (Thorpe et al. 2020).

Transcription factors (TFs) are at the core of this regulatory landscape. TFs orchestrate the expression of plant defense genes by binding to cis-regulatory DNA elements (CREs) within complex gene regulatory networks (GRNs) (Zaret 2020). Their modular architecture allows mutations and domain rearrangements contributing to the functional and structural diversity (Cheatle Jarvela and Hinman 2015). Upon perception of the external or internal stimuli, TFs are essential to integrate multiple signaling pathways leading to coordinated transcriptional responses (Tsuda and Somssich 2015; Yuan et al. 2024). Several TF families, including WRKY, MYB, bHLH, NAC, AP2/ERF, bZIP, TCP, HSF, and GRAS, play crucial roles in modulating immune responses, hormonal crosstalk, secondary metabolism, oxidative stress response, and cellular repair during herbivore attack (Pauwels and Goossens 2011; Hu et al. 2013; Xia et al. 2014; Song et al. 2017; Züst and Agrawal 2017). Numerous reports suggests that aphids can manipulate these TF networks through their salivary effectors. They reprogram the host transcriptional machinery to suppress plant defense and create a nutrient-rich environment conducive to their own growth and reproduction (Sandström et al. 2000; Mondal 2017).

Transcriptional reprogramming of the host raises a critical question- Do these aphid-induced transcriptional changes reflect plant-initiated resistance responses, or do aphids actively manipulate and takeover host cellular processes to create a more favourable environment for their own feeding and survival? Understanding the host-regulated defense activation and pest-driven reconfiguration of TF pathways remains one of the most critical challenges in modern plant-aphid molecular biology.

Given the rapid expansion of transcriptomic, genomic, and effector biology research, now there is a need to summarise current knowledge on how TFs shape plant responses to aphid colonization. This review provides a comprehensive overview of the plant defense response including hormonal regulation of immunity against aphids and TF families involved in aphid response, their regulatory mechanisms, including their integration with complex signaling pathways, along with the strategies aphids use to modulate these networks. We also highlight emerging genomics and functional tools, identify key knowledge gaps, and discuss opportunities for translating TF-based knowledge into durable aphid resistance in crops for improved pest management.

## Plant defense signaling pathways activated during aphid feeding

Aphid feeding activates a multilayered defense response in plants involving early perception around the stylet penetration site and systemic signaling networks that reprogram host transcriptional machinery (Jaouannet et al. 2014; Züst and Agrawal 2016; Gebretsadik et al. 2025). The first line of defense involves physical barriers such as trichomes, glandular hairs, and the cuticular wax layer. These barriers reduce aphid movement, penetration efficiency and settling (War et al. 2012; Fürstenberg-Hägg et al. 2013).

Upon successful stylet insertion, plants initiate different modes of immune responses. At cellular levels, PTI is activated when cell membrane localized, conserved pattern-recognition receptors (PRRs) detect herbivore-associated molecular patterns (HAMPs) or damage-associated molecular patterns (DAMPs) generated during the probing (Jaouannet et al. 2014; Prince et al. 2014; Gong et al. 2024). Although no canonical HAMPs have been identified from aphids, several aphid-associated molecular patterns including salivary proteins acts as elicitors to trigger PTI-like responses (De Vos and Jander 2009; Kumaraswamy and Huang 2024). Aphids secrete salivary effectors to counteract PTI by suppressing defense signaling, and maintain phloem sap accessibility (Naessens et al. 2015; Reymond and Calandra 2015; Wang et al. 2021; Xue et al. 2024; Liu et al. 2025). However, detection of these effectors and elicitors leads to the activation of a stronger and more specific immune response termed as ETI (Wang et al. 2023). Beyond these localized defense responses, plants can develop enhanced resistance through defense priming. A wide range of elicitors, herbivore-associated organisms (HAOs: fungi, bacteria, viruses), and HIPVs act as priming agents. These signals promote long-lasting systemic defense against subsequent aphid attacks (Zhu et al. 2014; Rashid et al. 2017; Javed et al. 2022; Gong et al. 2024; Liu et al. 2024).

**Fig. 1:**
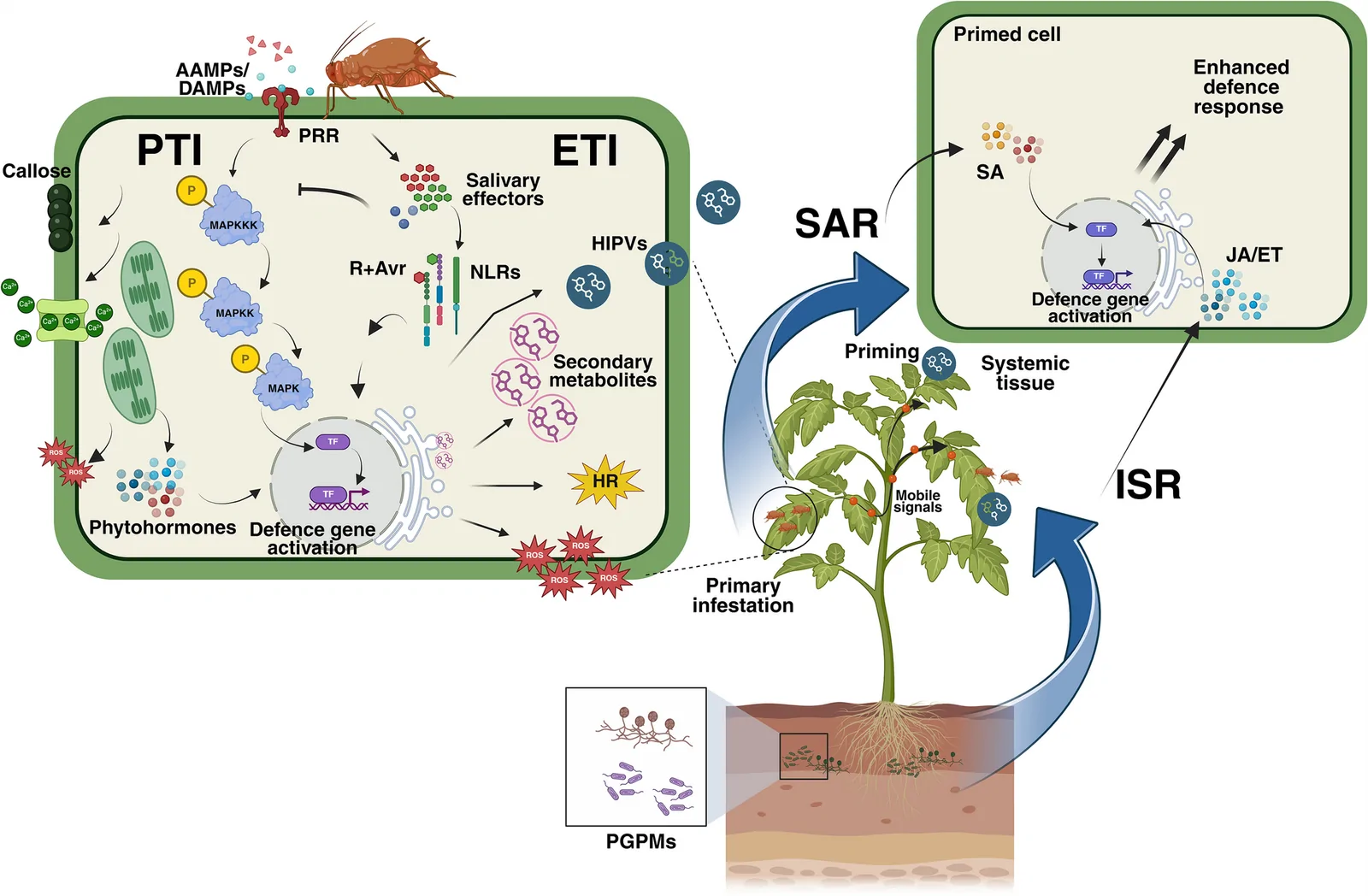
Plant defense signaling pathways activated during aphid attack. During aphid feeding, aphid-associated molecular patterns (AAMPs; e.g. *GroEL*) and damage-associated molecular patterns (DAMPs) such as oligogalacturonides are perceived by conserved pattern-recognition receptors (PRRs) like *BAK1* and *Wall-Associated Kinase 1 (WAK1)*, triggering a MAPK signaling cascade involving MPK1, MPK2, and MPK3. This signal relay leads to the activation of pattern-triggered immunity (PTI) and DAMP-triggered immunity (DTI). PTI responses include cytosolic Ca^2^⁺ influx, reactive oxygen species (ROS) production, induction of phytohormones such as salicylic acid (SA), jasmonic acid (JA), ethylene (ET), and abscisic acid (ABA), activation of defense-related genes, callose deposition for cell wall reinforcement, and accumulation of secondary metabolites including phenolics, flavonoids, tannins, lectins, and protease inhibitors. Aphid salivary effectors can suppress PTI to facilitate colonization; however, their recognition by intracellular nucleotide-binding leucine-rich repeat receptors (NLRs) activates a stronger effector-triggered immunity (ETI) response. Local defense signaling, together with herbivore-induced plant volatiles (HIPVs) and phloem-mobile signals, primes distal uninfested tissues, leading to a heightened and long-lasting defense response mediated predominantly by SA, termed systemic acquired resistance (SAR). In parallel, beneficial plant growth-promoting microorganisms (PGPMs; e.g. *Pseudomonas* spp. *Bacillus* spp.), including rhizobacteria and mycorrhizae, activate induced systemic resistance (ISR) through JA- and ET-dependent signaling pathways, enhancing protection against aphid attack

Central to these layered defense strategies are conserved signaling cascades involving MAPKs and core phytohormones such as salicylic acid (SA), jasmonic acid (JA), and ethylene (ET) (Seo et al. 1995; Morkunas et al. 2011; Hettenhausen et al. 2015; Patil and Nandi 2020; Manjeet and Yadav 2021; Yang et al. 2024). Additionally, other phytohormones such abscisic acid (ABA), gibberellic acid (GA) and reactive oxygen species (ROS) such as hydrogen peroxide (H_2_O_2_) and nitric oxide (NO) also regulate defense modulation and cross-talk (Shih et al. 2023). These defense- response pathways are depicted in Fig. 1. Together, signaling pathways form the biochemical and molecular framework within which transcription factors operate and coordinate downstream defense responses. Understanding these pathways is essential for deciphering how plants orchestrate resistance and how aphids manipulate them to promote susceptibility.

## Basal defense against aphids- Pattern-Triggered Immunity (PTI) and Effector-Triggered Immunity (ETI)

Evolutionary conserved, surface localised PRRs comprising of receptor kinases (RKs) or receptor-like proteins (RLPs), enable plants to detect conserved molecular patterns shared among diverse insect, fungal, and bacterial pathogens (Zipfel 2014; DeFalco and Zipfel 2021). These patterns, collectively termed pathogen-, microbe-, herbivore- or damage-associated molecular patterns (PAMPs/MAMPs/HAMPs or DAMPs), form the basis of PTI response (Choi and Klessig 2016).

Aphids lack the classical HAMPs like β-glucosidase, caeliferins, inceptin, volicitin, and fatty acid amides (FAAs), which are commonly found in chewing insects (Mattiacci et al. 1995; Alborn et al. 1997; Yoshinaga et al. 2007; Carroll et al. 2008). They induce basal immunity through elicitors present in their saliva (Jaouannet et al. 2014). For example, green peach aphid (GPA; *M. persicae)* feeding or salivary infiltration locally activates SA/JA/ET-independent defense response in Arabidopsis via a 3 to 10 kDa elicitor protein, inducing genes such as *O-methyltransferases* without increasing deterrent glucosinolate levels (De Vos and Jander 2009). Whole body extract of the GPA also activate *BRASSINOSTEROID INSENSITIVE1-ASSOCIATED KINASE1 (BAK1)-*mediated PTI in Arabidopsis, including upregulation of the camalexin biosynthetic gene, *PHYTOALEXIN DEFICIENT3 (PAD3)* (Prince et al. 2014). Consistently, the potato aphid *(Macrosiphum euphorbiae)* saliva carries GroEL protein which is a *Buchnera aphidicola* endosymbiont derived chaperonin. GroEL is delivered into the host plants during aphid feeding and acts as a molecular pattern that induces *BAK1*-dependent PTI signaling, leading to defense gene activation and reduced aphid fecundity (Chaudhary et al. 2014).

In response to PTI, insects secrete a repertoire of effectors to perturb host defense by interfering with the PTI machinery (Wang et al. 2023). Aphids secrete effectors via watery saliva to manipulate plant defenses and facilitate colonization (Table 1), while gelling saliva forms a protective stylet sheath during the feeding (Tjallingii 2006; Will et al. 2007; Hogenhout and Bos 2011; Mugford et al. 2016; van Bel and Will 2016; Bleau et al. 2024).

**Table 1:** Aphid salivary effectors and associated proteins involved in modulation of plant defense responses during aphid-plant interactions

Sr. No	Effector/Salivary Protein	Aphid Species	Reported Function During Plant Interaction	Reference
1	Ap4	*Acyrthosiphon Pisum*	Increases aphid fecundity by targeting the host BPL proteins	(Shih et al. 2025)
2	C002	*Acyrthosiphon Pisum*	Essential for aphid feeding and survival on host plants	(Mutti et al*.* 2008)
3	Armet	*Acyrthosiphon Pisum*	Knockdown shortens aphid lifespan and affects survival	(Wang et al*.* 2015)
4	ACPISUM_029930 (ApCHL1)	*Acyrthosiphon Pisum*	Putative avirulence-associated effector that binds chitin	(Lopez-Cobollo et al*.* 2026)
5	ApMIF1–ApMIF5	*Acyrthosiphon Pisum*	Suppress callose deposition, hypersensitive response, and defense transcripts	(Dubreuil et al*.* 2014)
6	ACE1 and ACE2	*Acyrthosiphon Pisum*	Facilitate aphid feeding and survival	(Wang et al. 2015)
7	AcE4	*Aphis citricidus*	Inhibits INF1/BAX-induced chlorosis in *N. benthamiana*	(Shangguan et al*.* 2024)
8	AcDCXR (diacetyl/L-xylulose reductase)	*Aphis craccivora*	Putative effector involved in energy generation and defense modulation	(MacWilliams et al*.* 2020)
9	Mp1, Mp58, Me10, sheath proteins	*Aphis craccivora*	Involved in digestion, detoxification, and modulation of plant defenses	(Pavithran et al*.* 2024)
10	Me10 and Me23	*Aphis euphorbiae*	Enhance aphid fecundity	(Atamian et al. 2013)
11	Me47	*Macrosiphum euphorbiae*	Functions as both elicitor and suppressor of plant defenses	(Kettles and Kaloshian 2016)
12	MsEF1	*Melanaphis sorghi*	Activates RMES1A/RMES1B-mediated resistance in sorghum	(Lei et al. 2024)
13	CathB3	*Myzus persicae*	Binds EDR1-like protein to trigger ROS accumulation and suppress phloem ingestion	(Guo et al. 2020)
14	CathB6 (cathepsin B)	*Myzus persicae*	Recruits immune regulators to suppress plant defense responses	(Liu et al*.* 2025)
15	C002, PIntO1 (Mp1), PIntO2 (Mp2)	*Myzus persicae*	Enhance aphid fecundity and virulence	(Pitino and Hogenhout 2013)
16	Mp1	*Myzus persicae*	Promotes aphid colonization by targeting VPS52 and disrupting host vesicle trafficking	(Rodriguez et al*.* 2017)
17	Mp55	*Myzus persicae*	Suppresses plant defense responses	(Elzinga and Jander 2013)
18	Mp10 and Mp42	*Myzus persicae*	Trigger plant defense responses and reduce aphid fecundity	(Bos et al. 2010)
19	Mp58	*Myzus persicae*	Forms a complex with Mp1 and interacts with VPS52 to facilitate infestation	(Bleau et al*.* 2026)
20	Mp10 and Mp42	*Myzus persicae*	Activate SA- and JA-mediated defenses, reducing aphid reproduction	(Rodriguez et al*.* 2014)
21	miR29-b	*Myzus persicae*	Suppresses host defense via cross-kingdom RNA interference targeting BAG4	(Han et al*.* 2025)
22	MpCP2	*Myzus persicae*	Binds cellulose and reduces release of cell wall-derived DAMPs	(Tong et al*.* 2026)
23	MpMIF1, MpMIF3, MpMIF4	*Myzus persicae*	Repress cryptogein-induced hypersensitive response	(Naessens et al. 2015)
24	CarD763 (carotenoid desaturase)	*Myzus persicae*	Promotes feeding and colonization by enhancing host photosynthesis	(Ge et al*.* 2024)
25	Carbonic anhydrase II (CA-II)	*Myzus persicae*	Alters apoplastic pH and promotes vesicle trafficking and virus spread	(Guo et al*.* 2023)
26	Mp14-3–3	*Myzus persicae*	Interacts with NtABI5 and modulates defense gene expression	(Du et al*.* 2025)
27	Mp64	*Myzus persicae*	Targets SIZ1 and enhances host susceptibility	(Liu et al*.* 2022)
28	Mp56, Mp57, and Mp58	*Myzus persicae*	Activate SA- and JA-mediated defenses, negatively affecting aphid reproduction	(Elzinga and Jander 2013)
29	Rp1 and Rp58	*Rhopalosiphum padi*	Cooperative effector proteins involved in host manipulation	(Bleau et al*.* 2026)
30	RpC002 and Rp1	*Rhopalosiphum padi*	Suppress barley defense responses and enhance susceptibility	(Escudero-Martinez et al*.* 2020)
31	Sg2204	*Schizaphis graminum*	Suppresses wheat defense responses and facilitates feeding	(Zhang et al*.* 2022a)
32	SaCDK (cyclin-dependent kinase-like protein)	*Sitobion avenae*	Suppresses BAX- and INF1-triggered cell death	(Zhang et al. 2024a)
33	SaApo AI (apolipoprotein AI)	*Sitobion avenae*	Modulates SA–JA crosstalk and suppresses hypersensitive responses	(Zheng et al*.* 2026)
34	Sm9723	*Sitobion miscanthi*	Suppresses INF1-induced programmed cell death	(Zhang et al*.* 2022b)
35	SmCSP4	*Sitobion miscanthi*	Activates SA-mediated defense through interaction with TaWRKY76	(Zhang et al. 2023b)
36	Sm10 and SmC002	*Sitobion miscanthi*	Suppress callose deposition and modulate SA-mediated defenses	(Fu et al*.* 2023)

Some of these effectors suppress plant immunity and lead to effector- triggered susceptibility (ETS). For instance, MpC002, C002, PIntO1 (Mp1), and PIntO2 (Mp2) enhance *M. persicae* colonization on specific hosts, illustrating effector adaptation to host specificity (Mutti et al. 2006; Bos et al. 2010; Pitino and Hogenhout 2013). Mp55 and the endosymbiont-derived GroEL suppress glucosinolate, callose, and ROS responses, promoting *M. persicae* reproduction, while silencing Mp55 reduces aphid fitness (Elzinga et al. 2014). A cysteine protease Cathepsin B3 (CathB3) from *M. persicae* triggers ROS accumulation via tobacco EDR1-like kinase, limiting phloem feeding and colonization (Guo et al. 2020). Similarly, Cathepsin B (CathB) proteins from *M. persicae* recruit EDS1, PAD4, and ADR1 to plant processing bodies to suppress defenses. However plant HSP family protein Acd28.9 promote resistance by countering CathB activity (Liu et al. 2025). Me10 and Me23 from *M. euphorbiae* increase aphid fecundity by suppressing defenses in tobacco and tomato (Atamian et al. 2013; Chaudhary et al. 2015). Macrophage migration inhibitory factor (MIF1) and Sm9723 from the Indian grain aphid, *Sitobion miscanthi* inhibit defense gene expression, callose deposition, and hypersensitive cell death, contributing to survival, fecundity, and feeding, highlighting potential RNAi targets (Naessens et al. 2015; Zhang et al. 2022). Similarly, SmCSP4 from *S. miscanthi* interacts with *TaWRKY76* in wheat. This interaction downregulates the activation of SA-degrading gene *DMR6*, enhancing SA accumulation and deters aphid feeding (Zhang et al. 2023a). Ap4 from *Acyrthosiphon pisum* interacts with two BPL (Binding Partner of ACD11-Like) proteins, PsBPL1/2 in pea to enhance fecundity (Shih et al. 2025).

Conversely, some effectors trigger plant defenses, reducing aphid performance. Mp10 and Mp42 induce defense responses and reduce fecundity, with Mp10 additionally suppressing flg22-induced oxidative bursts (Bos et al. 2010). Mp10 also binds the acrostyle in *A. pisum* and *M. persicae*, facilitating effector delivery into plant cells and positively influencing feeding behavior (Deshoux et al. 2022). DAMP-triggered immunity (DTI) involves oligogalacturonides (OGs), which are suppressed by *M. persicae* during feeding. Mp10/CSP4 inhibits OG-induced defenses while modulating EDS1-mediated responses to enhance aphid fecundity and host adaptation (Gravino et al. 2025).

Detection of these effectors by intracellular nucleotide-binding (NB) leucine-rich repeat (LRR) receptors (NLRs; also called R-proteins) is central to ETI (Jones and Dangl 2006; Boissot 2023). The tomato resistance gene, *Mi* confers isolate-specific resistance against the potato aphid, *M. euphorbiae* and nematodes, representing the first cloned R gene effective against distantly related organisms and belonging to the NBS-LRR family (Rossi et al. 1998). In apple, the *Sd-1* and *Sd-2* loci have been identified as resistance genes against rosy leaf-curling apple aphid, *Dysaphis devecta* (Cevik and King 2002a, 2002b). Lettuce *RGC2* gene cluster encodes multiple resistance genes including *Dm3, Dm14, Dm16, Dm18* and *Ra,* against lettuce root aphid *(Pemphigus bursarius L.)*. Among these, *RGC2B* encodes *Dm3* gene which recognises Avr3 effectors to activate ETI (Wroblewski et al. 2007). Similarly, *RAP1* was identified as a novel R gene for resistance against the pea aphid *A. pisum* in *Medicago truncatula* (Stewart et al. 2009).

The melon *VAT* gene encodes a CC-NBS-LRR protein mediating dual resistance to *Aphis gossypii* infestation and aphid-transmitted viruses (Dogimont et al. 2014). Mi-1.2 in tomato provides race-specific resistance against phloem-feeding herbivores but also negatively affects non-target beneficial insects like *Orius insidiosus*, highlighting broader ecological impacts (Pallipparambil et al. 2014). Two important R gene loci, *Rag6* and *Rag3c* were identified from soybean conferring resistance against *Aphis glycines* (Zhang ShiChen et al. 2017). In wheat, resistance to the specialist aphid *Diuraphis noxia* is mediated by the NLR gene *Adnr1*, which contains C-terminal ankyrin repeats and an N-terminal WRKY domain; silencing *Adnr1* abolishes *Dn1*-mediated resistance, resulting in susceptibility and higher aphid reproduction (Nicolis and Venter 2018). Elevated CO₂ enhanced resistance to pea aphids (*Acyrthosiphon pisum)* in *M. truncatula* by upregulating HSP90, its co-chaperones, the JA signaling pathway, and ubiquitin-mediated proteolysis, whereas *A. pisum* performance increased on the *A17*-R-gene-lacking plants, highlighting HSP90’s role in ETI-mediated resistance (Sun et al. 2018). Interestingly, an aphid effector Armet, when delivered into plant tissues, activates MAPK- and SA-associated defense pathways, elevates salicylic acid levels, and enhances resistance to *Pseudomonas syringae* without harming aphid performance, revealing a unique aphid-specific effector function that triggers pathogen-like defenses and highlights a novel tripartite insect-plant-pathogen interaction (Cui et al. 2019).

In sorghum, RMES1A and RMES1B proteins confer defense against *Melanaphis sorghi* by interacting with the aphid effector MsEF1 in exocysts, triggering ROS bursts. RMES1A/B carry ATP-binding sites and leucine-rich-repeat domains, representing a novel type of resistance protein (Lei et al. 2024). In peach, three dominant genes viz*., Rm1, Rm2,* and *Rm3* mediate resistance to *M. persicae*. among four TIR-NBS-LRR genes, TNL1 is highly overexpressed in resistant clones, while TNL2 shows moderate induction, providing candidate genes for marker-assisted selection (Duval et al. 2022). The *Rm3* locus was fine-mapped to a 160 kb interval on chromosome 1, revealing two novel TIR-NLR genes, with NLR1 expressed specifically in resistant leaves and co-segregating with resistance, establishing it as the likely candidate gene (Pan et al. 2021).

Effector delivery relies on watery saliva and specialized structures like the acrostyle, which transiently binds effectors for cytoplasmic entry (Deshoux et al. 2022). Importantly, PAMPs and effectors often overlap in function and conservation, the classical divide between PTI and ETI is not absolute, and plant immunity is better viewed as a continuum driven by immune receptors recognizing diverse ligands to achieve effective defense (Thomma et al. 2011).

## Systemic acquired and induced resistance against aphids

Beyond local responses, a previously challenged or primed plant initiates a swift, long-distance signaling that enhances immune competence, a phenomenon known as systemic acquired resistance (Conrath 2006; Fu and Dong 2013). SAR is associated with the accumulation of mobile signals such as Salicylic acid (SA), methyl salicylate (MeSA), Jasmonic acid (JA), methyl jasmonate (MeJA), pipecolic acid (Pip), N-hydroxy Pipecolic acid (NHP), azelaic acid (Aza), and ROS waves, which prime distal tissues for faster and stronger responses to subsequent attacks (Singh et al. 2013, 2023; Gao et al. 2015; Patil and Nandi 2022).

Examples of SAR induction include volatile-mediated activation, where 3-pentanol and 2-butanone triggered resistance in cucumber against *M. persicae* by enhancing natural enemy recruitment through oxylipin-pathway activation (Song and Ryu 2013). Similarly, exogenous SA application in wheat strengthened defense enzymes and phenolic compounds, reducing *S. avenae* fecundity, growth, and survival (Feng et al. 2021), and methyl salicylate (MeSA) exposure in barley induced systemic and surface-level resistance, altering plant volatiles and reducing *Rhopalosiphum* *padi* settling and feeding (Ninkovic et al. 2021). Prior aphid infestations also trigger SAR-like responses in a host- and genotype-dependent manner. For example, cotton, wheat, and peach showed varied resistance to subsequent aphid attacks depending on plant genotype and aphid species (Wool and Hales 1996; Sauge et al. 2006; Liu et al. 2025).

In parallel, Induced Systemic Resistance (ISR) is primarily triggered by plant growth-promoting microbes (PGPMs), including rhizobacteria and fungi, which activate both SA- and JA/ET-dependent signaling pathways and constitute a key defense strategy against herbivory (Ramamoorthy et al. 2001; Choudhary et al. 2007; Van der Ent et al. 2009; Yu et al. 2022). For example, *Bacillus amyloliquefaciens* primes broad beans against *A. pisum*, reducing aphid reproduction, while the aphid endosymbiont *Hamiltonella defensa* can counteract ISR to benefit aphids (Serteyn et al. 2020). *Bacillus subtilis* 26D induces resistance in wheat against *R. padi* and *Schizaphis graminum* (Rumyantsev et al. 2023), whereas *Bacillus velezensis* YC7010 activates the PAD4-mediated defense pathway in Arabidopsis against *M. persicae* without altering SA, JA/ET, or ABA signaling (Rashid et al. 2017). In tomato, different strains of *Beauveria bassiana* elicit resistance against *M. persicae* (Basit et al. 2024), and synthetic microbial communities comprising *Brevibacterium frigoritolerans*, *Bacillus niacin*, *Soilbacillus silvestris*, and *Bacillus luciferensis* produce 1-nonanol, protecting pepper plants against aphids (Lee et al. 2025). *Azospirillum brasilense* also induces systemic resistance in wheat against *R. padi* (Santos et al. 2025).

Microbial protein elicitors can similarly induce ISR and defense activation. For example, PeaT1, PeBL1, Hrip1, and PeBA1 suppressed *Brevicoryne brassicae* on cabbage by activating JA, SA, and ethylene signaling and modifying leaf traits like trichomes (Hameed et al. 2024). The Hrip1 protein reduced colonization, development, and reproduction of *Megoura japonica* on *Phaseolus vulgaris* while enhancing JA, SA, and ethylene levels and altering plant growth traits (Javed et al. 2022).

Interestingly, not all microbe-induced responses are beneficial to plants. *Pseudomonas fluorescens* enhanced performance of the generalist aphid *M. persicae* in Arabidopsis by suppressing ABA signaling, without affecting the specialist aphid *B. brassicae*, illustrating that PGPR can also make plants more susceptible (Pineda et al. 2012). Aphid infestation modulates pepper root exudation and selectively recruits gram-positive rhizobacteria like *Paenibacillus polymyxa.* Aphids exploit this modification to increase own colonization (Kim et al. 2016).

SAR and ISR provide plants with systemic defense capabilities against aphids and other pests, mediated by chemical signals, microbial interactions, and genotype-dependent induced responses, highlighting the potential for exploiting these mechanisms in pest management strategies.

## Hormonal regulation of plant defense against aphids

Plant defense against aphids is tightly regulated by a network of phytohormones that perceive attack, integrate signaling cascades, and coordinate downstream transcriptional and metabolic responses. Among these, salicylic acid (SA), jasmonic acid (JA), ethylene (ET) and abscisic acid (ABA) are the primary regulators of herbivore-induced defense (De Vos et al. 2007; Browse and Howe 2008; Morkunas et al. 2011; Yang et al. 2024). These phytohormones modulate the intensity and specificity of the defense response in conjugation with several components including, transcription factors, MAPKs, ROS and other hormones such as, gibberellic acid (GA), auxins, and cytokinin (Howe and Jander 2008; Morkunas et al. 2011; Erb et al. 2012; Wang et al. 2016; Yang et al. 2024).

### Salicylic acid (SA) and Methyl Salicylate (MeSA)

SA and its methyl conjugate MeSA play a key role in plant defense against biotrophs and hemi-biotrophs (Spoel and Dong 2024). SA is mainly associated with the regulation of *Pathogenesis related (PR)* genes and development of systemic immunity in plants (Shah and Zeier 2013; Carella et al. 2014; Gao et al. 2015).

Involvement of SA signaling in the defense regulation against aphids (*M. persicae*) is widely reported from model plants like Arabidopsis,* M. truncatula* and *Nicotiana* (Moran and Thompson 2001; Kuśnierczyk et al. 2008; Donovan et al. 2013; Silva-Sanzana et al. 2022), as well as in different crops such as tomato, cotton, wheat, barley, sorghum and zucchini (Mohase and van der Westhuizen 2002; Li et al. 2006; Coppola et al. 2018; El-Sherbeni et al. 2019; Feng et al. 2021; Ninkovic et al. 2021; Pant and Huang 2022; Hu et al. 2023; Sorg et al. 2025).

For example, Russian wheat aphid *(D. noxia)* infestation selectively elevates SA and peroxidase activity in resistant wheat while inhibiting catalase in both resistant and susceptible plants (Mohase and van der Westhuizen 2002). Similarly, Arabidopsis responds to cabbage aphid feeding with rapid ROS and calcium signaling, activation of SA and JA pathways, callose deposition, glucosinolate remodeling, and camalexin accumulation (Kuśnierczyk et al. 2008). In tomato, Mi-1 gene-mediated defense against potato aphids depends on SA and MAPK cascade signaling (Li et al. 2006). Exogenous application of SA enhances defense response in susceptible wheat plants after *S. avenae* infestation (Feng et al. 2021) and prior aphid attack in zucchini primes SA-mediated responses that alter *A. gossypii* dispersal without affecting its development and reproduction (Coppola et al. 2018). Additionally, exogenous application of SA enhanced resistance of tea (*Camellia sinensis* cv. Qiancha1) plants against *Toxoptera aurantii* (Jiang et al. 2023). SA also regulates production of aphid-repellent compounds such as (E)-β-farnesene in chrysanthemum (Li et al. 2019a; Wang et al. 2025b).

Transcription factors also play a critical role in SA-mediated defense. For example, GhMYB18, an R2R3 MYB transcription factor from cotton, positively regulates SA and phenylpropanoid-mediated defenses against *A. gossypii* (Hu et al. 2023). SA can also enhance insecticidal activity when combined with profenofos (El-Sherbeni et al. 2019).

In addition, volatile derivative like MeSA from *Vicia faba* was identified as a repellent for black bean aphid, *Aphis fabae* Scop. (Hardie et al. 1994). Similarly, *R. padi* feeding behaviour and settling was also reduced on barley plants exposed to MeSA (Ninkovic et al. 2021). SA biosynthetic *PAL* genes in sorghum (*SbPAL*) are induced by sugarcane aphid infestation and significantly enhance SA-dependent resistance to reduce aphid population growth and fecundity (Pant and Huang 2022). Oligogalacturonides (OGs) application in Arabidopsis strengthen SA-mediated resistance to *M. persicae* by reducing the number of offsprings and altering its feeding behaviour (Silva-Sanzana et al. 2022).

Overall, these studies highlight that SA signaling is a central component of plant defense against aphids, orchestrating a wide range of molecular, biochemical, and physiological responses. SA-mediated defenses act both locally and systemically, involve transcriptional reprogramming, reactive oxygen species, secondary metabolite production, and volatile emission, and can be modulated by exogenous application or aphid effectors, underscoring the adaptability of SA-dependent aphid resistance.

### Jasmonic acid (JA)

Jasmonic acid (JA) is a central phytohormone traditionally associated with defense against chewing herbivores (Wang and Wu 2013). However, it also contributes significantly to aphid resistance by regulating secondary metabolites, proteinase inhibitors, and antioxidant defenses (War et al. 2012; Züst and Agrawal 2017). Aphids often manipulate JA signaling through salivary effectors to suppress plant defenses and maintain phloem sap accessibility (Naessens et al. 2015; Wang et al. 2021). JA-mediated responses are coordinated by MYC, MYB, and bHLH transcription factors, integrating signals into transcriptional reprogramming for both local and systemic defense.

For instance, bluegreen aphid *(Acyrthosiphon kondoi* Shinji) resistance in *M. truncatula* is mediated by a dominant gene that uniquely activates the octadecanoid (JA-biosynthetic) pathway in resistant plants (Gao et al. 2007). In *Brassica oleracea*, seed coating with JA prime long-term defenses, reducing the growth and reproduction of the aphid *M. persicae* (Haas et al. 2018). In wheat, PeaT1 protein enhances resistance to *S. avenae* by increasing JA-mediated physical defenses such as trichomes and wax deposition (Li et al. 2020). Furthermore, exogenous JA application enhances wheat growth, photosynthetic pigments, and antioxidant enzyme activities under aphid stress, reducing aphid populations by 60%–73% (Aslam et al. 2022).

In aphid-resistant soybean, jasmonic acid-isoleucine (JA-Ile) accumulation is induced by avirulent aphids but suppressed by virulent aphids, indicating that JA-Ile is a key phytohormone mediating resistance against *A. glycines* and virulent aphids may overcome resistance by avoiding JA-Ile induction (Yates-Stewart et al. 2020). Similarly, *A. gossypii* infestation in cucumber triggers JA, ET, and ROS signaling, and exogenous JA reduces aphid reproduction while upregulating JA-biosynthesis genes (Qi et al. 2020). Pre-infestation of citrus by *Aphis spiraecola* activate jasmonic acid (JA)-dependent defenses that reduce *Diaphorina citri* feeding and reproduction. Whereas *Aphis citricidus* suppress JA signaling and enhances *D. citri* performance (Gao et al. 2023).

In sorghum, jasmonic acid (JA) plays a dual role in defense against sugarcane aphid *(Melanaphis sacchari),* initially deterring aphid settling but later promoting aphid feeding and proliferation (Grover et al. 2022b). When *Schizaphis graminum* attack sorghum plants, the plants trigger a complex defense system involving 82 key genes related to stress and defense signalling. Further it was confirmed that the JA-regulated defense signalling was more potent against these greenbug infestations (Zhu-Salzman et al. 2004). Interestingly, constitutive JA induction in Arabidopsis reduces aphid growth via antibiotic effects rather than feeding deterrence (Kuśnierczyk et al. 2011). Upon herbivory by *S. littoralis*, Arabidopsis initiates a robust Jasmonic Acid (JA)-mediated defense response. This process is coordinated by a suite of transcription factors MYC2, MYC3, and MYC4 which function as a transcriptional complex to activate downstream defense genes (Schweizer et al. 2013; Schmiesing et al. 2016). Virus-mediated suppression of JA and JA-Ile by *A. pisum* virus enhances aphid survival (Lu et al. 2020). Tomato resistance to potato aphid is primarily JA-mediated, with glandular trichomes enhancing defense (Blanco-Sánchez et al. 2023). Furthermore, in tobacco, long glandular trichomes promote JA-enhanced defenses through diterpenoid accumulation, while non-glandular trichomes contribute via defensive proteins, showing tissue-specific roles of JA (Wang et al. 2022b).

### Ethylene (ET)

Ethylene (ET) often acts in conjunction with JA to fine-tune plant defenses against aphids, particularly in regulating antixenotic responses. Potato aphid feeding induces ET in tomato. Blocking ET reduces susceptibility in compatible plants without affecting Mi-1-mediated resistance (Mantelin et al. 2009). *S. graminum* induces ET in barley, triggering hydrogen peroxide accumulation and peroxidase activity as part of oxidative defense (Argandoña et al. 2001).

Ethylene signaling, activated by harpin, drives green peach aphid resistance in Arabidopsis by requiring key components like ETR1 and EIN2, making ET the central hormonal pathway during harpin-induced insect defense (Dong et al. 2004). ET is also strongly activated during resistant interactions in tomato and melon, including *M. euphorbiae* and *A. gossypii*, with ERF integrators like *Cm-ERF1* playing a central role (Anstead et al. 2010). Turnip mosaic virus (TuMV) increases green peach aphid reproduction by suppressing callose defenses in Arabidopsis. The viral protein NIa-Pro alters ethylene signaling and manipulates plant defenses to benefit its aphid vector (Casteel et al. 2015). In maize, ET regulates resistance to *Rhopalosiphum* *maidis* via the Mir1-Cys protease. This induces antibiosis and antixenosis responses and mediates long-distance root signaling (Louis et al. 2015).

However, Cucumber mosaic virus (CMV) infection in pepper (Capsicum annuum) increases ethylene production, which attracts aphid vectors *M. persicae* and *A. glycines*. Chemical inhibition of ethylene reduces aphid preference, while ethylene treatment of healthy plants increases attractiveness. Thus, ethylene acts as a volatile signal enhancing aphid attraction to virus-infected plants (Kwon et al. 2022). Similarly, In tomato, *ERF Pti5* limits potato aphid survival together with the *Mi-1.2* gene, with induction independent of ET, which primarily affects aphid settling (Wu et al. 2014). Arabidopsis* MYB102* is induced by *M. persicae*, increasing ET production, reducing resistance, and suppressing callose deposition (Zhu et al. 2018). ET signaling, often in conjunction with JA, orchestrates a multifaceted defense network against aphids. This includes transcriptional reprogramming, secondary metabolite production, ROS activation, physical defenses, and modulation of other hormones such as SA and ABA. JA and JA-ET responses are dynamic, tissue-specific, and influenced by environmental and biotic factors, demonstrating the sophisticated regulatory mechanisms plants employ to resist aphid infestation.

### Abscisic acid (ABA)

Abscisic acid (ABA) is primarily known for its role in abiotic stress responses, but it also plays a significant part in modulating plant defenses against aphids by interacting with other phytohormones and regulating stomatal closure, trichome density, and secondary metabolites.

In *Arabidopsis*, green peach aphid feeding induces ABA, which promotes aphid performance by suppressing defense compounds like indole glucosinolates. During Russian wheat aphid (*Diuraphis noxia*) infestations, wheat plants having *Dnx* resistant gene showed activation of ABA and ET responsive pathways compared to the susceptible *Dn0* plants (Smith et al. 2010). ABA-deficient plants show increased resistance and reduced aphid populations (Hillwig et al. 2016). In soybean, *A. glycines* exploit ABA to suppress SA and JA-mediated defenses (Studham and MacIntosh 2013; Hohenstein et al. 2025), enhancing aphid colonization and facilitating other herbivores, while ABA-deficient plants show increased JA signaling and reduced aphid populations (Hohenstein et al. 2025).

In *M. truncatula*, drought-induced activation of the ABA reduces SA-mediated defenses but enhances JA-mediated defenses. It further alters plant water status and impacts pea aphid *(A. pisum)* feeding and fecundity, showing how ABA signaling integrates abiotic stress with herbivore defense (Guo et al. 2015). In Arabidopsis, interactions between ABA and ascorbate-redox signaling during *M. persicae* infestation modulate defense responses, with ABA negatively affecting resistance while altered redox enhances defense and reduces aphid fecundity (Kerchev et al. 2013). Exogenous ABA can enhance aphid resistance in several crops. In rice, ABA treatment increases trichome density to reduce *S. avenae* feeding, and induces pest-related volatiles (Liang et al. 2024).

ABA signaling thus represents a double-edged sword. It can support tolerance by modulating growth and water relations but may also be exploited by aphids to circumvent plant defenses.

### Gibberellic acid (GA)

Gibberellic acid (GA) is classically associated with growth regulation, but it also participates in plant-aphid interactions, particularly in gall-inducing systems. *Schlechtendalia chinensis* induces horned galls on Chinese Sumac. Early in gall development, tannin accumulation peaks as GA rises. Later, GA decreases as aphid populations expand. Interestingly, exogenous GA accelerates gall development, indicating GA’s role in controlling gall growth while aphids manipulate GA to promote gall formation (Wang et al. 2016).

### Crosstalk among phytohormones

Plant defense against aphids is orchestrated by a complex network of interacting phytohormones (Morkunas et al. 2011). JA and ET act synergistically to enhance defenses, while other phytohormones like, ABA and GA can modulate JA and SA signaling depending on environmental conditions or aphid strategies (Wang et al. 2016; Hua et al. 2023). SA and JA interactions are often antagonistic (Schwartzberg and Tumlinson 2014; Schmiesing et al. 2016), synergistic interactions have also been reported (Schweiger et al. 2014; Selig et al. 2016). Aphids can manipulate this crosstalk through salivary effectors or virus-mediated regulation, including suppression of JA signaling or ET enhancement that attracts vectors (Lu et al. 2020; Kwon et al. 2022).

In Arabidopsis, cabbage aphid feeding rapidly activates SA and JA signaling around stylet punctures (Rubil et al. 2022). Studies in sorghum provide detailed evidence of coordinated activation of multiple signaling pathways. Aphid infestation increases JA, SA, ABA, and auxins in resistant genotype Tx2783, while exogenous application of SA, JA, and ABA enhances resistance in the susceptible BTx623 (Huang et al. 2022). Transcriptomic analyses of the Tx2783 show the upregulation of genes associated with SA/JA pathways, early signal perception, and defense-related metabolism after sugarcane aphid attack. Exogenous SA or JA further reduces aphid damage (Shrestha et al. 2024). A variety of other plant-aphid systems illustrate similar interactions. In peach, aphid (*Tuberocephalus momonis*) feeding triggers BAP accumulation alongside JA, ABA, and ACC/ET responses, ultimately contributing to gall abscission (Hua et al. 2023).

Regulatory elements like TFs also shape plant susceptibility. *WRKY22* suppresses SA- and JA-mediated defenses to promote *M. persicae* performance, while *wrky22* mutants show enhanced resistance (Kloth et al. 2016). In *Plantago lanceolata*, separate JA and SA treatments modify metabolites to decrease aphid survival, whereas combined JA and SA weakens these effects due to antagonistic signaling (Schweiger et al. 2014). Feeding by pea aphids on *Vicia faba* suppresses JA while inducing SA, exemplifying antagonistic interactions (Schwartzberg and Tumlinson 2014). Spraying of MeJA moderately affected *S. miscanthi* behaviour but strongly activated JA-responsive genes while suppressing SA pathways in wheat, highlighting the antagonistic hormonal crosstalk that shapes cereal defenses during aphid infestation (Yan et al. 2023). Similarly, *Therioaphis trifolii* resistant strains manipulate host defenses by using saliva to activate SA signaling and suppress JA responses, thereby reducing plant repellency and enhancing aphid survival on resistant alfalfa cultivars (Zhu et al. 2024). Whereas in wheat, *Sitobion avenae* salivary effector *SaCDK* suppresses both SA- and JA-mediated defenses, reduces callose deposition, and enhances aphid survival and fecundity, thereby improving aphid host adaptation (Zhang et al. 2024a).

Additional studies show how hormone crosstalk regulates both direct and indirect defenses. In Arabidopsis, insect egg deposition activates SA signaling that suppresses JA-mediated defenses through MYC2/3/4 degradation and *WRKY75* regulation (Schmiesing et al. 2016). In *M. truncatula*, JA and SA responses differ between chewing and piercing herbivores, although downstream responses resemble pathogen-induced signaling (Leitner et al. 2005). Experiments involving exogenous hormone treatments further emphasize the complexity of this regulatory network. In soybean, SA application alone does not influence aphid performance, but SA combined with MeJA significantly reduces populations, underscoring JA’s dominant role (Selig et al. 2016). In pepper, *M. persicae* feeding induces early JA and JA-Ile accumulation, followed by SA and ABA modulation (Florencio-Ortiz et al. 2018).

Environmental conditions also modulate hormone-based defenses. Elevated CO₂ increases *M. persicae* populations in Arabidopsis by enhancing SA levels while suppressing JA and JA/ET signaling pathways (Sun et al. 2013). Temporal coordination of signaling is also evident in pea, where aphid infestation triggers early JA accumulation followed by later SA, ET, and NO induction (Mai et al. 2014). Sugarcane aphid tolerance in the sorghum SC35 genotype is associated with higher basal JA precursor levels, controlled accumulation of SA, ABA and feeding-induced cytokinin, enabling the plant to maintain growth and photosynthesis despite high aphid populations (Grover et al. 2022a).

Additionally, The tolerant soybean genotype KS4202 exhibits higher constitutive ABA and JA levels and related transcript expression, and responds to prolonged aphid infestation by upregulating peroxidases, indicating that phytohormone-based constitutive defenses and ROS detoxification contribute to its tolerance to soybean aphids (Chapman et al. 2018). The dynamic crosstalk ensures that plants can balance immediate defense, long-term tolerance, and growth, while aphids evolve strategies to manipulate this network to their advantage.

## Transcription factors in plant defense against aphids

Transcription factors (TFs) are central regulators of plant gene expression, controlling diverse physiological, developmental, and stress-responsive processes (Hrmova and Hussain 2021; Dhatterwal et al. 2024; Kaleh et al. 2024; Alamos et al. 2025). TFs coordinate the activation or repression of downstream genes by binding to specific promoter regions, enabling plants to rapidly respond to the stimuli (Schreiter and Drennan 2007; He et al. 2023; Koyama 2024). In the context of biotic stress, TFs play a crucial role in integrating signaling from multiple phytohormones and environmental cues, fine-tuning defense responses to maximize resistance while minimizing growth penalties (Kajla et al. 2023; Bhoite et al. 2025).

During aphid attack, plants face a unique challenge due to the piercing-sucking feeding mode of these insects. Aphids elicit localized and systemic responses that involve rapid signaling through phytohormones (Rubil et al. 2022; Gebretsadik et al. 2025). TFs act as essential mediators of these signaling networks, translating hormonal cues into specific transcriptional programs that regulate defense gene expression, secondary metabolite production, and structural modifications such as callose deposition or lignification (Duhlian et al. 2020; Koch et al. 2020; Saha et al. 2023). Moreover, TFs form complex regulatory networks with other TFs, coactivators, and repressors, integrating multiple signaling pathways and environmental factors to produce a coordinated defense outcome (Bhati et al. 2025; Gray and Grotewold 2025). Aphids can manipulate these networks through salivary effectors or virus-mediated modulation, suppressing defense-related TF activity and enhancing host susceptibility (Zhang et al. 2017). Understanding the roles of different TF families in these processes is therefore essential for elucidating the molecular basis of plant-aphid interactions and for developing strategies to improve crop resistance.

### WRKY transcription factors

WRKY transcription factors are major regulators of plant defense, rapidly activated after herbivore attack and functioning at key nodes of hormonal, MAPK, and Ca^2^⁺-mediated signaling networks (Pandey and Somssich 2009; Hu et al. 2015; Yan et al. 2018; Li et al. 2024a). By binding W-box elements *(TTGACC/T)*, WRKY TFs fine-tune downstream defense gene expression and orchestrate responses such as secondary metabolite production, ROS regulation, and cell wall reinforcement (Brand et al. 2010; Jiang et al. 2014; Phukan et al. 2016; Chu et al. 2025; Li et al. 2025; Mahanta et al. 2025).

WRKY TFs play a key role in plant defense against aphids. In Arabidopsis, 16 WRKY genes were rapidly induced by *B. brassicae*, with WRKY75 showing the strongest upregulation at 48 hpi (Kuśnierczyk et al. 2008). Similarly, WRKY induction has been reported in *M. truncatula* during Bluegreen aphid *(A. kondoi)* infestation (Gao et al. 2010) and in chrysanthemum leaves attacked by *Macrosiphoniella sanbourni* (Xia et al. 2014). In wheat, early infestation by *S. graminum* upregulated WRKYs along with SA and MAPK signaling genes (Zhang et al. 2020b).

Additionally, Several WRKYs contribute to aphid resistance in diverse crops. *WRKY70, WRKY72a/b* are essential for *Mi-1*-mediated defense in tomato and Arabidopsis (Bhattarai et al. 2010; Atamian et al. 2012). Furthermore, Arabidopsis WRKY70 mediates density-dependent crosstalk between *B. brassicae* and *Plutella xylostella*, where low aphid density elevates *WRKY70* to suppress JA defenses and aid caterpillars, while high density lowers *WRKY70*, boosts *MYC2*, and strengthens JA-mediated resistance (Kroes et al. 2014). *CmWRKY48* in chrysanthemum is induced by aphid, *M. sanbourni* and its overexpression reduces aphid growth and shows enhanced resistance (Li et al. 2015). *RlWRKY10/14* are strongly induced by *Macrosiphum rosae* feeding in rose (Zhu et al. 2022). *WRKY75*, *WRKY40* are induced in aphid resistant rose cultivars (Dong et al. 2024). Tobacco *NtWRKY28*, induced by *M. persicae*, enhances resistance via activation of phenylpropanoid and lignin biosynthesis (Chu et al. 2025).

Furthermore, aphids actively modulate WRKY-mediated defense signaling across multiple crops including cotton, wheat, soybean, and alfalfa. For example, the wheat aphid salivary protein SmCSP4 suppresses *TaWRKY76* to enhance host SA accumulation (Zhang et al. 2023a). In cotton, *WRKY33* and *WRKY3* are consistently upregulated during *A. gossypii* feeding, while *WRKY1/20/21* are downregulated (Dubey et al. 2013). Soybean WRKY transcription factors, including *GmWRKY11*, exhibit genotype-specific responses to Soybean aphid infestation (Yao et al. 2020). Pyramiding *Resistance to* *Aphis* *glycines* *(Rag1/2)* resistance genes further amplifies this response, with WRKYs highly enriched among *Rag1/2*-specific differentially expressed genes at 6 h (13 of 23 WRKYs expressed exclusively in the pyramid line), underscoring their central role in *Rag* genes-mediated defense (Natukunda et al. 2021). Consistently, tolerant soybean genotypes upregulate multiple WRKYs upon aphid attack, including orthologs of Arabidopsis WRKYs known to regulate SA-JA crosstalk and general stress responses, highlighting their importance in soybean resistance to *A. glycines* (Prochaska et al. 2015). Beneficial microbes also influence WRKY-mediated defense. *Rhizophagus intraradices* enhances alfalfa resistance to pea aphid by elevating *WRKY* gene expression, antioxidant activity, and SA levels (Li et al. 2019b). Similarly, nitrate-modulated aphid resistance in tobacco involves *WRKY14, WRKY40,* and *WRKY41* (Miao et al. 2025).

Whole-genome microarray analysis in Arabidopsis revealed that *M. persicae* induces several *WRKY* TFs, most notably *WRKY53* and *WRKY62*, which function in redox- and SA-mediated signaling and act downstream of *NPR1* to regulate SA-dependent defense against aphids (Kerchev et al. 2013). *WKRY53* also plays crucial role in resistance against *D. noxia* infestation in wheat as Virus-induced gene silencing (VIGS) of *WKRY53* conferred susceptibility to wheat plants and improved aphid performance (Van Eck et al. 2010). Several WRKY TFs were upregulated in sorghum resistant lines upon sugarcane aphid infestation (Serba et al. 2021). GWAS analyses in sorghum identified *SbWRKY86* as a major locus for sugarcane aphid resistance and revealed additional WRKYs, LRRs, LOXs, and flavonoid-pathway genes associated with resistance to *M. sorghi* (Poosapati et al. 2022; Punnuri et al. 2022).

Conversely, *WRKY22* negatively regulates resistance in Arabidopsis by modulating SA-JA crosstalk during *M. persicae* infestation (Kloth et al. 2016), and *CmWRKY53* suppresses secondary-metabolite defense genes in chrysanthemum against *Macrosiphoniella sanborni* (Zhang et al. 2020a), demonstrating that WRKYs can also act as negative regulators.

Collectively, these findings demonstrate that WRKY transcription factors shape plant responses to aphid attack in highly dynamic and context-dependent ways. WRKYs integrate signals from multiple defense networks to fine-tune resistance outcomes. Their recurrent involvement across diverse host-aphid systems points to a conserved regulatory role, making WRKYs valuable breeding candidates and promising leverage points for developing durable aphid resistance in crops.

### MYB transcription factors

MYB transcription factors constitute one of the largest and functionally diverse families of plant transcription regulators. They are defined by the presence of a highly conserved MYB DNA-binding domain, typically consisting of 1 to 4 imperfect repeats of approx. 50–54 amino acids, each forming three α-helices that fold into a helix-turn-helix (HTH) structure (Frampton et al. 1991; Ogata et al. 1994; Wang et al. 2019a). The second and third helices of each repeat mediate specific DNA recognition, primarily binding to cis-regulatory elements such as the MYB-binding site in target gene promoters (Martin and Paz-Ares 1997; Dubos et al. 2010). The variable C-terminal domain is responsible for transcriptional activation or repression (Wu et al. 2024; Tatar Caliskan et al. 2025). Based on the number of repeats, plant MYBs are classified into R1-MYB, R2R3-MYB, R1R2R3-MYB, and 4R-MYB families, with R2R3-MYBs being predominant and widely involved in regulating plant secondary metabolism and defense responses (Kaur et al. 2010; Du et al. 2012; Ambawat et al. 2013; Chezem et al. 2017).

Consistent with their established roles in plant defense, several MYB genes are rapidly activated during aphid attack. For example, In *M. truncatula*, early resistance to *A. kondoi* and *A. pisum* involves rapid induction of MYB transcription factors in resistant lines, whereas susceptible plants show suppressed expression (Gao et al. 2010). Similarly, chrysanthemum transcriptomes show strong MYB activation upon *M. sanbourni* feeding (Xia et al. 2014). In peach, MYB transcription factors contribute to *Rm3*-mediated resistance against *M. persicae* through regulation of hormone, redox, and secondary metabolite pathways (Niu et al. 2018). MYB TFs are also differentially expressed in *T. trifolii f. maculata*-resistant lines (Jacques et al. 2020). In soybean, MYB TFs regulate *A. glycines* resistance by modulating JA and SA- dependent defenses (Yao et al. 2020).

In Arabidopsis, MYB15 is induced by *M. persicae* and functions in ABA- and redox-mediated defense (Kerchev et al. 2013), while *AtMYB15, AtMYB38,* and *AtMYB44* are induced by bacterial hairpin protein and participate in ethylene-mediated defense against green peach aphid (Liu et al. 2010, 2011). *AtMYB44* further enhances resistance to green peach aphid by activating EIN2-dependent ethylene signaling and promoting phloem-based and glucosinolate defenses (Lü et al. 2013). Overexpression of *AtMYB12* in tobacco increases flavonoid content and strengthens defense against *M. persicae* through ROS, H₂O₂, and NO production (Ding et al. 2021).

In wheat, *TaMYB19, TaMYB29,* and *TaMYB44* activate phloem-based defenses against English grain aphid by inducing callose synthases and phloem lectins (Zhai et al. 2017), while *TaMYB31* enhances resistance to *R. padi* under drought and aphid stress by regulating benzoxazinoid biosynthesis and callose deposition (Hao et al. 2025).

Another R2R3-MYB TF, *GhMYB18* in cotton enhances resistance to *A. gossypii* by activating SA and flavonoid pathways and boosting defense enzyme activities (Hu et al. 2023). In rose, *MYB108* is strongly induced in aphid-resistant cultivars (Dong et al. 2024). R2R3-MYBs like *BjA06.GL1* and *BjB02.GL1* regulate trichome formation in *Brassica juncea*, and CRISPR/Cas9 knockouts lead to glabrous, *Lipaphis erysimi* susceptible leaves (Heng et al. 2024).

In chrysanthemum, *CmMYB19* and *CmMYB15* are induced by *M. sanbourni* feeding and activate lignin-biosynthetic genes to enhance lignin deposition and restrict aphid multiplication (Wang et al. 2017; An et al. 2019). The SG2-type R2R3-MYB *CmMYB15-like* activates *Cm4CL2* to promote lignin accumulation and strengthen cell walls under aphid attack (Li et al. 2023a). Additionally, *CmMYBML1* enhances resistance by activating the *CmTGA1-CmTPS1* cascade and promoting sesquiterpene biosynthesis in response to aphid feeding (Wang et al. 2025a).

In Arabidopsis, AtMYB102 is strongly induced by *M. persicae*. It increases ethylene biosynthesis by upregulating *ACS* genes and suppresses callose-based defenses, thereby enhancing host susceptibility (Zhu et al. 2018).

Overall, MYB transcription factors function as central regulators that integrate metabolic and defense pathways during aphid infestation. Their rapid induction in resistant plants, together with their regulation of secondary metabolism, ROS homeostasis, and defense gene expression, highlights their pivotal role in enabling effective responses against aphid attacks.

### NAC Transcription factors

NAC (NAM, ATAF1/2, and CUC2) transcription factors are a large, plant-specific family with a conserved N-terminal NAC domain (~150 amino acids) for DNA binding and dimerization, and a variable C-terminal transcriptional regulatory region (TRR) for transcriptional modulation (Fang et al. 2008; Kim et al. 2024). NAC TFs regulate diverse biological processes, including growth and development, organ senescence, hormonal signaling, and responses to biotic and abiotic stresses (Zhong et al. 2011; Diao et al. 2020; Chen and Xia 2025; Xin et al. 2025; Zhang et al. 2025a). Functionally, NAC TFs integrate signals from JA, SA, ET, and ABA hormones with downstream transcriptional machinery to regulate cell wall modification, ROS homeostasis, and defense gene expression against insect herbivory (Bu et al. 2008; Yoshii et al. 2010; Bian et al. 2021; Eerapagula et al. 2025; Gu et al. 2025; Zhao et al. 2025).

In legume and cucurbits, NAC TFs mediate early defense against aphids. In *M. truncatula*, resistance to Bluegreen aphid involves early induction of NAC TFs in AKR resistant gene containing genotypes (Gao et al. 2010). Similarly, in resistant melon lines with the *Vat* R gene, *NAC22* and *NAC100* homologs are regulated by *miR164.* Thes NAC TFs are specifically upregulated after *A. gossypii* feeding, highlighting miRNA-mediated NAC defense (Sattar et al. 2012, 2016). ROS-mediated defenses link NAC regulation to aphid resistance. In maize, *R. padi* and *S. avenae* herbivory activates the ascorbic acid -glutathione (AsA-GSH) antioxidant system more strongly in resistant genotypes, while reduced GSH levels in Arabidopsis* pad2-1* mutants suppress NAC TF expression and compromise defense (Sytykiewicz 2016). In *B. juncea*, infestation by the successful aphid *L. erysimi* triggers strong NAC-mediated transcriptional responses, whereas the unsuccessful aphid *Aphis craccivora* induces weaker NAC responses, indicating NACs are key regulators in compatible interactions (Duhlian et al. 2020). In transgenic tobacco, NAC TFs are upregulated in low ascorbate oxidase (AO) plants during aphid perception (Rasool et al. 2017). Whereas, in nitrate-modulated tobacco-aphid interactions, NAC TFs connect cell wall and lignin pathways with JA/SA signaling to enhance resistance (Miao et al. 2025).

Soybean NAC TFs contribute to both tolerance and resistance. *NAC19* shows higher constitutive expression in aphid-tolerant genotypes and is rapidly induced at early infestation to mediate ABA-dependent signaling (Chapman et al. 2018). Furthermore, long-term *A. glycines* infestation regulates PTI and phenylpropanoid/isoflavonoid defenses by inducing *NAC090, NTL9-like,* and *NAC036* TFs (Hohenstein et al. 2019). In *Rag1/2* pyramid genotypes, *NAC, WRKY,* and *JAZ* TFs are highly represented among synergistically induced TFs (Natukunda et al. 2021). *GmASMT* genes interact with NAC and other TFs to regulate development, stress responses, and aphid resistance (Kumar et al. 2022).

In cereals, NAC TFs play important role during aphid defense. Sorghum NAC TFs are strongly induced in response to sugarcane aphid and greenbug, reducing aphid population growth, with nine *SbNAC* genes specifically induced in resistant genotypes. Furthermore, NACs constitute a part of the regulatory network controlling *SbJAZ* genes and JA-mediated defenses (Kiani and Szczepaniec 2018; Shrestha et al. 2024; Zhang and Huang 2024). In maize, NAC TFs are upregulated in resistant lines during *R. maidis* and *R. padi* feeding, coordinating defense and benzoxazinoid regulation (Song et al. 2017; Pingault et al. 2021). MeSA treatment primes defense by upregulating 30 NAC TFs and enhances SA, flavonoid, and benzoxazinoid pathways (Li et al. 2024b). Similarly, in moderately resistant *Aegilops tauschii*, NAC TFs are induced in response to *R. padi*, activating ROS-scavenging and SA/ET signaling to reduce aphid survival (Narang et al. 2025).

Trichoderma treatment primes tomato against *M. euphorbiae* by inducing NAC and other TFs, alongside transcriptomic and metabolomic changes that enhance direct and indirect resistance (Coppola et al. 2019). NAC transcription factors, along with WRKY and other key TFs, are implicated in regulating aphid resistance in rose (Dong et al. 2024). Moreover, aphid-resistant peach genotypes induce NAC and other TFs, along with triterpenoid biosynthesis, to strengthen defense against green peach aphids (Pan et al. 2024). Thus, NAC transcription factors emerge as central regulators of plant defense across diverse species, integrating hormonal and ROS signals, coordinating with WRKY, MYB, and JAZ TFs, and modulating downstream pathways to confer both constitutive and inducible resistance against aphids.

### AP2/ERF Transcription factors

The APETALA2/ethylene response factor (AP2/ERF) superfamily is a diverse and significant plant TF family. AP2/ERF TF is characterized by a highly conserved AP2 DNA-binding domain (DBD) made-up of approx. 60 to 70 amino acids (Phukan et al. 2017; Feng et al. 2020). Based on these DBDs, AP2/ERF superfamily is divided into five subfamilies viz*.,* AP2, ERF, RAV (related to *Abscisic acid insensitive3/Viviparous1*; ABI3/VP1), DREB *(Dehydration Response Element Binding)*, and soloist (Sakuma et al. 2002; Tang et al. 2024). AP2/ERF TFs regulates multiple processes including flowering, development, metabolism, senescence, drought-, cold-stress responses, and hormone signaling (Elliott et al. 1996; Alonso et al. 2003; Dubouzet et al. 2003; Woo et al. 2010; Müller and Munné-Bosch 2015; Li et al. 2017; He et al. 2025). AP2/ERFs also integrate hormone signaling, MAPK cascades, and downstream defense responses during biotic stress, including insect herbivory (Lu et al. 2011; Meng et al. 2013; Nakayasu et al. 2018; Wu et al. 2020; Cai et al. 2023; Su et al. 2025).

Aphid resistance in Arabidopsis involves JA and ET signaling activating ERFs such as *AtERF11* and *AtERF13*, contribute to defense against aphids such as *M. persicae* and *B. brassicae* (Liu et al. 2010; Kuśnierczyk et al. 2011; Barah et al. 2013). Similarly, in Chrysanthemum, *M. sanbourni* feeding induces *ERF* transcription factors involved in SA-JA/ET signaling, ROS regulation, and secondary metabolite pathways (Xia et al. 2014). Grafted chrysanthemum shows strong upregulation of nine *ERF* genes, with four exhibiting particularly high expression during ET/JA- mediated defense signaling (Zhang et al. 2019b). Additionally, the *ERF* transcription factor *CmHRE2-like* is induced by aphid infestation and contributes to plant susceptibility, likely by downregulating flavonoid biosynthesis, whereas repression of *CmHRE2-like* enhances aphid resistance (Wang et al. 2024).

Other crops like melons, tomatoes and cabbage also highlight the role of ERFs in aphid defense. In melon, *cmERF1* plays a key role in *Vat*-mediated defence response involving ET signaling against *A. gossypii* (Anstead et al. 2010). Tomato ERF, *Pti5* mediates ethylene-independent antibiotic defense against potato aphid, acting synergistically with *Mi-1.2* resistance (Wu et al. 2014), while *BrERF11b* in Chinese cabbage enhances resistance against aphids and chewing insects via transcriptional repression through the EAR motif (Wu et al. 2020). In *M. truncatula*, elevated CO₂ suppresses ET signaling and ERF expression during pea aphid infestation, reducing ROS-based defenses and enhancing aphid performance (Guo et al. 2014). ERF1 is induced by aphid feeding and AM colonization, highlighting its role in tripartite aphid–plant-AM fungus interactions (Maurya et al. 2018). Similarly, ERF transcription factor (Medtr4g008860) is upregulated in the aphid-resistant line upon *T. trifolii f. maculata* feeding, indicating its key role in aphid resistance (Jacques et al. 2020).

Rm3-mediated resistance in peach, involves early induction of ERF, WRKY, and MYB TFs, MAPK, and phytohormone pathways (Niu et al. 2018). ERFs are also induced by mustard aphid in *B. juncea* (Duhlian et al. 2020) and by soybean aphid in *Glycine max*, acting through JA in susceptible and antixenotic genotypes and SA in antibiotic genotypes (Yao et al. 2020). *Macrosiphum rosae* L. infestation in rose, induces AP2/ERF transcription factors, along with bHLH, MYB, NAC, and WRKY, suggesting a key role for ERFs in mediating defense responses and enhancing resistance in tolerant cultivars (Dong et al. 2024). In tobacco, aphid infestation under different nitrate (NO₃^−^) levels affects the expression of multiple transcription factors, including *ERF, bHLH, NAC,* and *WRKY*. *ERF* TFs are implicated in regulating defense responses, cell wall reinforcement, and hormone-mediated pathways (Su et al. 2025). In wheat, aphid infestation disrupts diurnal rhythmicity of genes enriched with *ERF* motifs, indicating a role for ERFs in modulating circadian rhythm and defense responses (Goldstein et al. 2025).

Overall, AP2/ERF transcription factors are key regulators of plant defense against aphids, integrating hormone signaling, ROS, and secondary metabolite pathways to modulate resistance or susceptibility. Studies in Arabidopsis, chrysanthemum, medicago, tomato, peach, soybean, and other crops consistently highlight ERFs as key components linking signal perception to transcriptional reprogramming and downstream defense responses. Their conserved roles across crops highlight their potential as targets for enhancing insect resistance and developing sustainable pest management strategies.

### bHLH Transcription factors

The basic helix-loop-helix (bHLH) transcription factors constitute the second largest TF family in plants (Feller et al. 2011). Their conserved C-terminal domain contains two α-helices separated by a loop that enables dimerization, while a basic N-terminal region mediates DNA binding to ‘CAN’ half sites within the E-box (CANNTG) motif (Ellenberger et al. 1994; Carretero-Paulet et al. 2010; Gordân et al. 2013; de Martin et al. 2021). bHLHs regulate diverse developmental and signaling processes and are central to drought, cold, and salt responses (Jiang et al. 2009; Moreno et al. 2018; Guo et al. 2021; Hao et al. 2021; Yang et al. 2021; Xu et al. 2022, 2023). Increasing evidence also highlights their roles in plant defense (Wang et al. 2019b; Bai et al. 2020; Meraj et al. 2020; Yan et al. 2024).

bHLH transcription factors are widely activated across plant-aphid interactions. Their upregulation has been reported in Arabidopsis challenged by *B. brassicae* (Barah et al. 2013), cotton responding to *A. gossypii* (Dubey et al. 2013; Zhong et al. 2022), in soybean responding to *A. glycines* feeding (Yao et al. 2020) and rose infested by *M. rosae* (Dong et al. 2024). Similar induction occurs in maize against the corn leaf aphid, *R. maidis* (Pingault et al. 2021) in sorghum where bHLHs contribute to *SbJAZ*-mediated defense during sugarcane aphid attack (Kiani and Szczepaniec 2018; Shrestha et al. 2024). Resistant *M. truncatula* lines also show strong induction of *bHLH-1* and *bHLH-2* up to ~33-fold higher than susceptible genotypes during Bluegreen aphid interactions (Gao et al. 2010). Similarly, *bHLH101* and related members are among the most responsive TFs in *B. juncea* exposed to *L. erysimi* and *A. craccivora* (Duhlian et al. 2020).

Network analyses in hybrid switchgrass further highlight *bHLH* enrichment in aphid-responsive modules, with a key *bHLH* in module M2 linked to ribosome and metabolic pathways associated with protein processing and amino acid biosynthesis (Koch et al. 2020).

A well-characterized example in cotton is the MYC2-like bHLH *GhMYC1374*, which is strongly induced by *A. gossypii* and JA-SA signaling; its overexpression enhances flavonoid and gossypol biosynthesis and confers aphid resistance, while silencing reduces these defenses (Zhang et al. 2023b).

Atypical bHLH *PRE* genes, including *SbPRE4*, also contribute to aphid resistance during sorghum-*M. sacchari* and Arabidopsis-*M. persicae* interactions (Guo et al. 2024). In tobacco, nitrate-dependent aphid infestation triggers the induction of several insect-responsive TFs, including the bHLH member *NtbHLH71*, indicating a role for bHLHs in coordinating nitrogen-linked defense and metabolic responses (Miao et al. 2025).

Overall, transcriptomic evidence across multiple plant-aphid systems clearly demonstrates that bHLH transcription factors are consistently activated during aphid attack, emphasizing their importance in defense regulation. However, despite these strong expression-based indications, the functional characterization of most aphid-responsive bHLHs remains limited, and the specific molecular mechanisms through which they modulate resistance are still largely unresolved. Future studies dissecting their regulatory networks, target genes, and crosstalk with hormonal pathways will be essential to fully understand their roles in plant-aphid interactions.

Several additional transcription factor families also contribute to plant responses to aphid herbivory beyond the commonly studied WRKY, MYB, NAC, ERF, and bHLH groups. Transcriptomic studies have identified *bZIP* factors as part of early signaling networks activated during aphid attack, while *C2H2* zinc-finger TFs are frequently differentially expressed and linked to stress-responsive gene regulation. *DOF, GRAS* and *HSF* transcription factors also appear in aphid-induced gene modules, reflecting roles in hormone crosstalk, protein homeostasis, and broader stress adaptation (Gao et al. 2010; Xia et al. 2014; Song et al. 2017; Serba et al. 2021). Together, these diverse TF families highlight the complex multilayered regulatory architecture governing plant-aphid interactions.

## Plant secondary metabolites (PSMs) and their role in plant defense against aphids

Plant secondary metabolites (PSMs) play a crucial role in plant defense against aphids in addition to physical barriers such as trichomes, thorns, and cuticular waxes, as well as molecular defense mechanisms regulated by phytohormones and transcription factors. Plants produce a diverse array of secondary metabolites such as alkaloids (Corcuera 1984; Ma et al. 2018, 2020), terpenoids (Gupta et al. 2017; Kroes et al. 2017), saponins (De Geyter et al. 2012; Roonjho et al. 2022), pyrethrins (Prota et al. 2014; Papanikolaou et al. 2018), glucosinolates (Ahmed et al. 2022; Loitongbam et al. 2024), phenols (Aly et al. 2022; Javed et al. 2025), flavonoids (Riddick 2024; Zhang et al. 2025b), lectins (Li et al*.* 2023b; Deeksha et al. 2024), lignin (Li et al. 2023a; Chu et al. 2025) and tannins (Rodríguez et al. 2022), which collectively contribute to resistance against aphid infestation.

PSMs function through both direct and indirect defense mechanisms. Direct defenses include toxicity, antifeedant activity, repellence, interference with aphid’s digestion, and reduced reproduction; whereas indirect defenses involve the attraction of aphid predators and parasitoids through herbivore-induced plant volatiles (HIPVs) (Farhan et al. 2024). Several essential oils (EOs) rich in secondary metabolites exhibit strong aphicidal and repellent activities. Plant derived essential oils (EOs) from *Mentha piperita, Mentha longifolia, Salvia officinalis*, and *Salvia rosmarinus*, contain several secondary metabolites having aphicidal, repellent and deterrent activities against *Aphis punicae* (Sayed et al. 2022). Essential oils and secondary metabolites like farnesol, geraniol, (E)- anethole and jasmone act as repellent against two aphid species viz., *M. persicae* and *M. euphorbiae* (Cantó-Tejero et al. 2022). Similarly, EOs containing volatile terpenes from *Mentha arvensis*, *Mentha piperita* and *Lavandula angustifolia* showed repellent properties against *A. gossypii* (Zhang et al. 2024b). Polygodial and pyrethrins act as antifeedant against *M. persicae, A. fabae* and *A. gossypii* (Prota et al. 2014; Papanikolaou et al. 2018; Korir et al. 2021). Notably, nano formulations of pyrethrins enhanced the insecticidal activities against *A. gossypii* without hampering the predator populations (Papanikolaou et al. 2018). Alkaloids represent another important class of aphid-resistant metabolites. Alkaloids isolated from *Sophora alopecuroides* L. showed aphicidal activities against *A. craccivora and A. pisum* (Ma et al. 2018, 2020). Alkaloids like stylopine, corydaline, demethylcorydalmine, isocorypalmine, glaucine, and pseudoprotopine showed insecticidal properties against *A. gossypii* (Park et al. 2011). Triterpene *Quillaja Saponaria* saponins, showed cytotoxic and aphicidal properties against *A. pisum* as the aphids fed on artificial diet containing saponins had aberrations on gut epithelial (De Geyter et al. 2012).

Plants also deploy specialized proteins to disrupt aphid physiology. The expression of specialized proteins, including lectins like PTA and AHA, and protease inhibitors such as cystatins, Bowman-Birk inhibitors; BBI, impairs aphid digestive physiology by interfering with protease activity and gut function, thereby diminishing aphid growth, fertility, and overall lifespan (Ibrahim et al. 2018; Khoshfarman-Borji et al. 2020). The lectin Phloem Protein2-A1 (PP2-A1) from Arabidopsis, when included in a synthetic diet, negatively impacted weight gain in *M. persicae* and *A. glycines* (Beneteau et al. 2010). Studies in *Brassica* and wild peach relative *Prunus davidiana*, illustrate that plants utilize several secondary metabolites to endure intense herbivory. Transcriptomic and metabolomic studies further reveal the complexity of PSM-mediated aphid defense. In the wild peach relative *P. davidiana*, the cytochrome P450 gene *PpCYP716A1* regulates production of betulin, an aphid-specific toxic metabolite that confers resistance without negatively affecting beneficial insects (Wang et al. 2022a). Sorghum plants with high levels of polyphenols were found less susceptible to *M. sacchari* (Knoll et al. 2021). In a similar way, the infestation of *A. gossypii* in cotton triggers the expression of the *GhMYB18* transcription factor, which enhances the production of salicylic acid (SA) and flavonoids resulting in increased plant resistance to aphids (Hu et al. 2023). In cotton the overexpression of the *(E)-β-caryophyllene synthase* gene (*GhTPS1*) significantly lowers *A. gossypii* populations (Zhang et al. 2019a). Resistance to rosy apple aphid, *Dysaphis plantaginea* is contributed by 4-caffeoylquinic acid (4-CQA) and 4-p-coumaroylquinic acid (4-pCoQA) in apple cultivars (Berrueta et al. 2018). Additionally, monoterpenes act as natural repellents against *M. persicae* by reducing the time aphids spend probing leaves (Dancewicz et al. 2020). The biosynthesis of many secondary metabolites is tightly regulated by transcription factors including MYC2, MYB28, MYB29, NAC, and WRKY proteins (Guo et al. 2018), which integrate JA- and SA-dependent signaling pathways to coordinate chemical defenses and reinforce structural barriers such as lignification (Huang et al. 2023). Experimental studies, ranging from gene expression to herbivore-performance assays, have demonstrated that the plants do not rely on the single strategy to protect themselves. Instead, they dynamically integrate both chemical defense and physical barriers to mitigate damage (Papazian et al. 2019; Yang et al. 2020). NPR1 and ETR1 help increase total glucosinolate levels when aphids feed on the plant (Mewis et al. 2006). TFs like MYB and bHLH regulate enzymes involved in the biosynthesis of deterrent compounds such as glucosinolates and alkaloids. For instance, when the gene CmMYB15 is overexpressed, it helps chrysanthemums resist aphids by boosting lignin production (An et al. 2023). Flavonoids such as quercetin not only protect plants from ultraviolet radiation and regulate auxin transport, but also exhibit strong aphid-repellent and insecticidal properties (Riddick 2021).

Aphid feeding also induces major changes in plants’ primary and secondary metabolism, including reduction in soluble sugars, proteins, and amino acids, accompanied by an elevation in lipid peroxidation and the release of herbivore-induced volatile organic compounds (VOCs) like (E)-ß-farnesene and other terpenes. These VOCs function as signals for self-defense and indirect defense by attracting natural predators of aphids, exemplifying an adaptive and diverse to survive biotic stress (Verheggen et al. 2013; Najar-Rodriguez et al. 2015). Volatiles produced during *A. pisum* feeding on *Vicia faba* attracted aphid parasitoid *Aphidius ervi* (Du et al. 1998). Hydrolyzed products of glucosinolates influence aphid parasitoid *Diaeretiella rapae* behaviour (Pope et al. 2008). Lab assays showed that the honeydew secreted from *M. persicae* and *A. gossypii* can attract the aphidopagous gall midge, *Aphidoletes aphidimyza* (Rondani) (Choi et al. 2004; Watanabe et al. 2016). Interestingly, these volatiles induced oviposition behaviour in *A. aphidimyza* on eggplants (Higashida et al. 2022). Another HIPV, (Z)−3-hexenyl propanoate [(Z)−3-HP], acts as a repellent to *A. solani* and attracts parasitoid*, Aphelinus abdominalis* (Depalo et al. 2022). *A. gossypii* infested cucumber and potato plants emit volatiles to attract predator ants *Formica pratensis* (Schettino et al. 2017). Furthermore, *A. gossypii* induced volatiles could attract predatory ladybird beetles, *Cheilomenes sexmaculata* (Fab.) and *Hippodamia variegata* in cotton (Yi et al. 2023; Yasa et al. 2024). Potato plants exposed to HIPVs transiently disrupted *M. persicae* aphid feeding and attracted its natural enemies like *Harmonia axyridis* and *Aphidius gifuensis* (Ali et al. 2026).

Collectively, these studies demonstrate that plant secondary metabolites constitute a highly dynamic and multilayered defense system against aphids. Plants integrate transcriptional regulation, hormone signaling, specialized metabolites, structural reinforcement, and volatile-mediated indirect defenses to minimize aphid colonization and feeding damage. The coordinated regulation of these pathways by transcription factors and phytohormone networks highlights the sophisticated nature of plant defense strategies. Understanding the biosynthesis, regulation, and ecological functions of PSMs not only advances our knowledge of plant-aphid interactions but also provides promising opportunities for developing sustainable aphid management strategies through metabolic engineering, defense priming, and breeding of resistant crop cultivars.

## Recent advancements in developing aphid-resistant plants

Modern aphid-resistance breeding relies on various emerging tools and technologies, including integration of wild genetic diversity, advanced molecular tools, and emerging insights into epigenetic regulation of plant defense. Wild and semi-wild crop relatives represent crucial reservoirs of resistance to aphids. Many of the resistance traits have been successfully introgressed into cultivated varieties. For example, aphid-resistance traits derived from wild germplasm have contributed to improved resistance in potato and chrysanthemum through conventional and trigenomic hybridization approaches (Deng et al. 2012; Ali et al. 2022) However, barriers such as self-incompatibility, linkage drag, and reactive oxygen species (ROS)-associated incompatibility responses can complicate introgression efforts. Recent advances in tissue culture, embryo rescue, and molecular breeding strategies are helping overcome these obstacles and facilitate efficient gene transfer (Zhang et al. 2021).

Beyond traditional breeding, genome-editing technologies such as CRISPR/Cas9 are enabling precise modification and de novo domestication of wild species while retaining their natural resistance traits. These approaches provide opportunities to improve agronomic characteristics, including yield and nutritional quality, without compromising inherent stress resilience. In parallel, growing evidence suggests that epigenetic mechanisms, including DNA methylation, histone modification, and small RNA-mediated regulation, contribute to defense priming and stress memory, thereby enhancing plant responsiveness to repeated aphid infestation (Wibowo et al. 2016; Annacondia et al. 2021).

Advances in quantitative trait locus (QTL) mapping, genome-wide association studies (GWAS), bulk segregant analysis (BSA), and multi-omics approaches have accelerated the identification of aphid-resistance genes and regulatory networks in crops such as maize, cotton, soybean, pea, and cucumber (Liang et al. 2016; Ollivier et al. 2022; An et al. 2023). In pea, the major-effect locus ApRVII, was shown to confer resistance against *A. pisum* (Ollivier et al. 2022). In addition, transgenic and protein-engineering approaches are further expanding aphid-management strategies (Van Damme 2021) For example, transgenic cotton expressing lectins from *Sclerotium rolfsii* reduced aphid populations by approximately 69% (Vanti et al. 2018),whereas the fusion proteins in *Brassica juncea* lower down the aphid survival to 40% (Javaid et al. 2018). Similarly CRISPR-mediated knockout of the sugar transporter gene *VST1* in watermelon reduced aphid-associated damage and improved resistance (Li et al. 2021).

Altogether, these advances demonstrate how modern breeding, genome engineering, and functional genomics are transforming the development of aphid-resistant crops. Integration of transcription factor-mediated defense regulation with precision breeding and biotechnology offers promising opportunities to develop high-yielding, stress-resilient cultivars while reducing dependence on chemical insecticides and supporting sustainable agricultural systems.

## Conclusion and future perspectives

Plants response to aphid herbivory is a complex process governed by highly coordinated network of components like phytohormones, signaling cascades and transcription factors. Understanding these complexities is essential to develop strategies that can be used to enhance plant resistance to aphids under variable climatic conditions. Recent advances in multi-omics approaches have significantly broadened our understanding of molecular mechanisms of aphid-plant interactions, starting from signal perception to downstream defense activation. Within this framework, enhanced production of secondary metabolites like flavonoids, tannins, lectins and protease inhibitors can lead to promising crop protection measures against aphids. Parallelly, development of elicitor and inducer like compounds are essential for systemic long-term defense response development and this aspect may be further explored using biochemical and metabolomic approaches.

At the regulatory level, transcription factors act as a central node of plant defense, coordinating multiple signaling pathways, secondary metabolism, and gene regulation to fine-tune plant responses to aphid herbivory. Members of important TFs families belonging to WRKY, MYB, NAC, ERF, and bHLH, have been implicated in promising defense strategies during aphid-plant interactions (Table 2). However, most functional insights are derived from controlled experimental conditions using model plants like arabidopsis, medicago, tobacco and a few crop plants such as cotton, wheat, sorghum, tomato, etc. Whereas many TFs involved in aphid resistance remain uncharacterized, particularly in non-model and horticultural crop species largely due to lack of stable transgenic development in higher plants. Moreover, functional redundancy and strong context dependency of transcriptional regulators pose additional challenges for their direct deployment in crop improvement.

**Table 2:** Transcription factors involved in plant defense responses against aphid herbivory

Sr. No	Transcription factor	TF family	Plant species	Aphid species	Regulatory trait during aphid interaction	Reference
1	WRKY11	WRKY	*Glycine max*	*Aphis glycines Matsumura*	Upregulated and associated with Rag-mediated resistance to soybean aphid	Yao et al. 2020; Natukunda et al. 2021; Prochaska et al. 2015
2	WRKY86	WRKY	*Sorghum bicolor*	*Melanaphis sorghi/M. sacchari*	GWAS/QTL candidate associated with field resistance to sugarcane aphid	Poosapati et al. 2022; Punnuri et al. 2022
3	WRKY53, WRKY56	WRKY	*Arabidopsis thaliana*	*Myzus persicae*	SA/redox-related defense regulator induced by aphids	Kerchev et al. 2013
4	WRKY53	WRKY	*Triticum aestivum*	*Diuraphis noxia*	Silencing of TaWRKY53 abolishes Dn1-mediated resistance and increases aphid reproduction	Van Eck et al. 2010
5	WRKY76	WRKY	*Triticum aestivum*	*Sitobion miscanthi*	Elevates SA and reduces aphid feeding	Zhang et al. 2023b
6	WRKY70	WRKY	*Solanum lycopersicum*	*Macrosiphum euphorbiae*	Required for Mi-1-mediated resistance to aphids and nematodes	Atamian et al. 2012; Bhattarai et al. 2010
7	WRKY70	WRKY	*Arabidopsis thaliana*	*Brevicoryne brassicae; Plutella xylostella*	Density dependent regulator of SA-JA crosstalk, modulating aphid and caterpillar performance:	Kroes et al. 2014
8	WRKY48	WRKY	*Chrysanthemum morifolium*	*Macrosiphoniella sanbourni*	Overexpression enhances lignification and resistance, reducing aphid growth	Li et al. 2015
9	WRKY53	WRKY	*Chrysanthemum morifolium*	*Macrosiphoniella sanbourni*	Negatively regulates secondary metabolite genes; promotes aphid susceptibility	Zhang et al. 2020a
10	WRKY28	WRKY	*Nicotiana tabacum*	*Myzus persicae*	Overexpression strengthens phenylpropanoid/lignin defenses and aphid resistance	Chu et al. 2025
11	WRKY15	WRKY	*Arabidopsis thaliana*	*Brevicoryne brassicae*	Broad WRKY activation during cabbage aphid feeding	Kuśnierczyk et al. 2008
12	WRKY75, WRKY40	WRKY	*Rosa* spp.	*Macrosiphum rosae*	Strongly induced in aphid resistant rose cultivars, contributing to resistance	Dong et al. 2024
13	WRKY70, WRKY72a, WRKY72b	WRKY	*Solanum lycopersicum; Arabidopsis thaliana*	*Macrosiphum euphorbiae*	Essential for Mi-1-mediated defense and related SA-JA regulatory nodes	Bhattarai et al. 2010; Atamian et al. 2012
14	WRKY10, WRKY14	WRKY	*Rosa* spp.	*Macrosiphum rosae*	Strongly induced by aphid feeding in rose, associated with resistance responses	Zhu et al. 2022
15	WRKY33, WRKY3	WRKY	*Gossypium hirsutum*	*Aphis gossypii*	Upregulated during aphid feeding, linked with induced defense	Dubey et al. 2013
16	WRKY1, WRKY20, WRKY21	WRKY	*Gossypium hirsutum*	*Aphis gossypii*	Downregulated during aphid feeding, potentially associated with susceptibility or reprogramming of defense	Dubey et al. 2013
17	WRKY11	WRKY	*Glycine max*	*Aphis glycines*	Shows genotype specific induction during infestation; enriched among Rag1/Rag2-specific DEGs, central to Rag-mediated resistance	Yao et al. 2020; Natukunda et al. 2021; Prochaska et al. 2015
18	WRKY14, WRKY40, WRKY41	WRKY	*Nicotiana tabacum*	*Myzus persicae*	Involved in nitrate-modulated aphid resistance, linking nutrition status to WRKY-mediated defense	Miao et al. 2025
19	WRKY22	WRKY	*Arabidopsis thaliana*	*Myzus persicae*	Negatively regulates resistance by modulating SA-JA crosstalk, promoting aphid performance	Kloth et al. 2016
20	MYB15	MYB	*Arabidopsis thaliana*	*Myzus persicae*	Play role in ABA- and redox-mediated defense	Kerchev et al. 2013
21	MYB15, MYB38, MYB44	MYB	*Arabidopsis thaliana*	*Myzus persicae*	Induce ethylene-mediated defense against green peach aphid	Liu et al. 2010; Liu et al. 2011
22	MYB44	MYB	*Arabidopsis thaliana*	*Myzus persicae*	Enhances resistance by activating EIN2-dependent ethylene signaling and promoting phloem-based and glucosinolate defenses	Lü et al. 2013
23	MYB19, MYB29, MYB44	R2R3-MYB	*Triticum aestivum*	*Sitobion avenae*	Activates phloem-based defenses by inducing callose synthase and phloem lectin genes, reducing aphid performance	Zhai et al. 2017
24	MYB31	R2R3-MYB	*Triticum aestivum*	*Rhopalosiphum padi*	Enhances resistance under combined drought and aphid stress by regulating benzoxazinoid biosynthesis and callose deposition	Hao et al. 2025
25	MYB18	R2R3-MYB	*Gossypium hirsutum*	*Aphis gossypii*	Positively regulates resistance by activating salicylic acid and flavonoid pathways and increasing defense enzyme activities	Hu et al. 2023
26	MYB12	R2R3-MYB	*Arabidopsis thaliana—*overexpressed in tobacco	*Myzus persicae*	Overexpression increases flavonoid content and strengthens defense	Ding et al. 2021
27	MYB108	MYB	*Rosa* spp.	*Macrosiphum rosae*	Strongly induced in aphid-resistant cultivars, indicating a role in resistance	Dong et al. 2024
28	BjA06.GL1, BjB02.GL1	R2R3-MYB	*Brassica juncea*	*Lipaphis erysimi*	Regulates trichome formation; CRISPR/Cas9 knockouts are glabrous and more susceptible	Heng et al. 2024
29	NAC22, NAC100	NAC	*Cucumis melo*	*Aphis gossypii*	Involve in miRNA mediated NAC defense	Sattar et al. 2012; Sattar et al. 2016
30	NAC19	NAC	*Glycine max*	*Aphis glycines*	Higher constitutive expression in aphid tolerant genotypes; rapidly induced early during infestation to mediate ABA-dependent signalling and tolerance	Chapman et al. 2018
31	NAC090, NTL9-like, NAC036	NAC	*Glycine max*	*Aphis glycines*	Induced during long term infestation; involved in PTI and phenylpropanoid/isoflavonoid defenses	Hohenstein et al. 2019
32	ERF11, ERF13	AP2/ERF	*Arabidopsis thaliana*	*Myzus persicae; Brevicoryne brassicae*	Activated by JA and ET signalling; contributes to defense against aphids as part of ERF network	Liu et al. 2010; Kuśnierczyk et al. 2011; Barah et al. 2013
33	HRE2-ike	AP2/ERF	*Chrysanthemum morifolium*	*Macrosiphoniella sanbourni*	Induced by infestation and promotes susceptibility, likely via downregulation of flavonoid biosynthesis; repression enhances resistance	Wang et al. 2024
34	ERF1	AP2/ERF	*Cucumis melo*	*Aphis gossypii*	Key component of Vat mediated defense; functions in ET signalling against cottonmelon aphid	Anstead et al. 2010
35	Pti5	AP2/ERF	*Solanum lycopersicum*	*Macrosiphum euphorbiae*	Mediates ethylene independent antibiotic defense; acts synergistically with Mi1.2 resistance gene	Wu et al. 2014
36	BrERF11b	AP2/ERF	*Brassica rapa/Chinese cabbage*	Aphids and chewing insects	Enhances resistance to aphids and lepidopteran herbivores via transcriptional repression	Wu et al. 2020
37	ERFs	AP2/ERF	*Medicago truncatula*	*Acyrthosiphon pisum*	Elevated CO2 suppresses ET signalling and ERF expression during infestation, reducing ROS based defenses and increasing aphid performance	Guo et al. 2014
38	ERF1	AP2/ERF	*Medicago truncatula*	*Acyrthosiphon pisum*	Induced by aphid feeding and arbuscular mycorrhizal colonization, indicating a role in tripartite aphidplantAM fungus interactions	Maurya et al. 2018
39	Medtr4g008860	AP2/ERF	*Medicago truncatula*	*Therioaphis trifolii f. maculata*	Upregulated in aphid resistant line; implicated as a key regulator of resistance	Jacques et al. 2020
40	MYC1374	bHLH (MYC2-like)	*Gossypium hirsutum*	*Aphis gossypii*	Strongly induced by aphids/JA/SA; overexpression increases flavonoids/gossypol and resistance, silencing reduces defense	Zhang et al. 2023c
41	bHLH1, bHLH2	bHLH	*Medicago truncatula*	*Acyrthosiphon kondoi*	linked to early defense signalling	Gao et al. 2010
42	bHLH71	bHLH	*Nicotiana tabacum*	*Myzus persicae*	Nitrate responsive bHLH induced by infestation; implicated in nitrogen linked defense	Miao et al. 2025
43	PRE4	bHLH	*Sorghum bicolor; Arabidopsis thaliana*	*Melanaphis sacchari; Myzus persicae*	Atypical bHLH modulating growth defense balance; contributes to resistance and affects aphid performance	Guo et al. 2024

Importantly, plant defense is inherently constrained by growth-defense trade-offs, where resource allocation toward defense responses can negatively impact growth and yield (Züst and Agrawal 2017). These trade-offs are often mediated by shared regulatory hubs, including hormone signaling and transcriptional reprogramming, which enable plants to fine-tune defense outputs depending on environmental context (Huot et al. 2014; Züst and Agrawal 2017). However, recent studies also suggest that these constraints are not absolute and can be mitigated through precise genetic and regulatory control, allowing partial decoupling of growth and defense (Wang and Wang 2014; Campos et al. 2016). In this context, transcription factors represent particularly attractive targets, yet their constitutive or misregulated expression may lead to undesirable pleiotropic effects on plant growth and productivity. Therefore, fine-tuning their spatial, temporal, or inducible expression is critical to maintaining an optimal growth-defense balance by minimizing fitness costs and avoiding yield penalties.

**Fig. 2:**
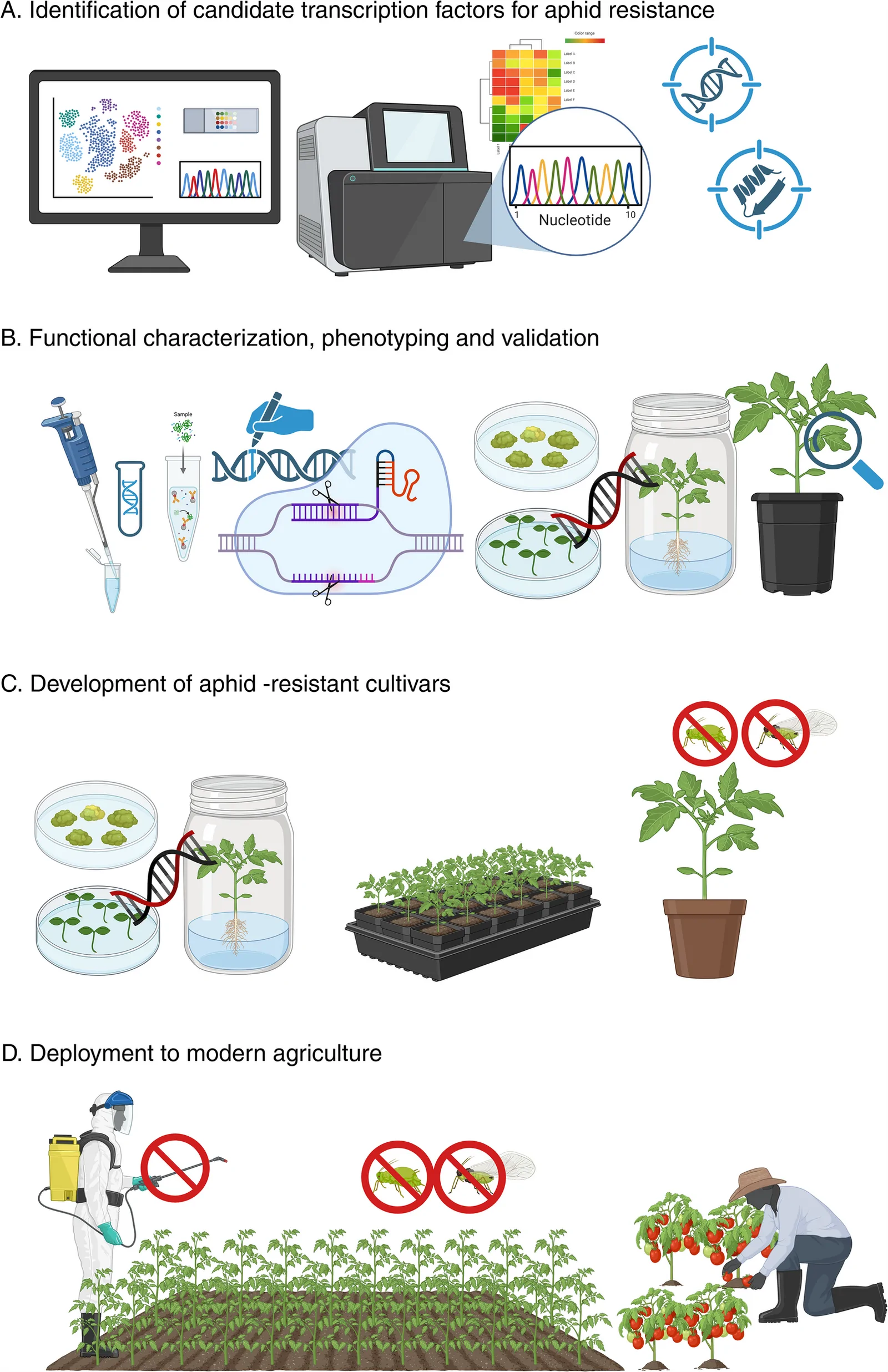
TF-mediated defense response induction as a translational strategy for aphid resistance. TF-mediated defense response induction can be explored to develop aphid resistant crops for sustainable pest management. **A** whole genome transcriptomic analyses with other omics approaches identify aphid-responsive TFs and regulatory hubs controlling hormone signaling, phloem defense, and secondary metabolism. **B** Functional characterization and screening of candidate TFs using advanced molecular techniques including ChIP-seq, CRISPR/Cas, VIGS, and transgenic approaches validate their roles in defense pathways. Phenotyping for aphid performance, feeding behavior, plant defense, and yield traits strengthen functional inference. **C** Validated TFs are integrated into marker-assisted breeding, genome editing, and defense-priming strategies to develop aphid-resistant cultivars. **D** Deployment of these aphid-resistant cultivars in modern agriculture mitigates aphid-induced crop losses, reduces pesticide reliance, and supports sustainable pest management

Future research should prioritize functional validation of these candidate TFs, using genome editing, RNAi, VIGS, and stable transformation systems to dissect their regulatory roles across multiple crops. Understanding host-aphid specificity and the extent of TF-mediated responses across diverse plant species will further strengthen our ability to identify key targets (Fig. 2). Enhanced understanding of these regulatory architectures will therefore be essential to develop crop varieties that maintain strong aphid resistance without compromising key agronomic traits such as growth, biomass, and yield stability under field conditions. In parallel, precision breeding strategies, including promoter engineering, inducible gene expression systems, and marker-assisted selection, offer promising avenues to decouple defense activation from yield penalties.

Collectively, integrating mechanistic insights from plant immunity with advanced molecular breeding tools will be crucial for translating fundamental discoveries into practical applications. Such approaches will enable the development of crop varieties that combine enhanced aphid resistance with stable yield and agronomic performance, thereby supporting sustainable agricultural systems under future environmental challenges.

## Data Availability

Not applicable.
